# An overview of the occurrence, impact of process parameters, and the fate of antibiotic resistance genes during anaerobic digestion processes

**DOI:** 10.1007/s11356-024-33844-3

**Published:** 2024-06-10

**Authors:** Kasra Pourrostami Niavol, Achinta Bordoloi, Rominder Suri

**Affiliations:** Department of Civil and Environmental Engineering, Temple University, Philadelphia, PA 19122 USA

**Keywords:** Anaerobic digestion (AD), Anaerobic co-digestion (AcoD), Antibiotic resistance genes (ARGs), Mobile genetic elements (MGEs), Antimicrobial resistance (AMR)

## Abstract

Antibiotic resistance genes (ARGs) have emerged as a significant global health threat, contributing to fatalities worldwide. Wastewater treatment plants (WWTPs) and livestock farms serve as primary reservoirs for these genes due to the limited efficacy of existing treatment methods and microbial adaptation to environmental stressors. Anaerobic digestion (AD) stands as a prevalent biological treatment for managing sewage sludge and manure in these settings. Given the agricultural utility of AD digestate as biofertilizers, understanding ARGs’ fate within AD processes is essential to devise effective mitigation strategies. However, understanding the impact of various factors on ARGs occurrence, dissemination, and fate remains limited. This review article explores various AD treatment parameters and correlates to various resistance mechanisms and hotspots of ARGs in the environment. It further evaluates the dissemination and occurrence of ARGs in AD feedstocks and provides a comprehensive understanding of the fate of ARGs in AD systems. This review explores the influence of key AD parameters such as feedstock properties, pretreatments, additives, and operational strategies on ARGs. Results show that properties such as high solid content and optimum co-digestion ratios can enhance ARG removal, while the presence of heavy metals, microplastics, and antibiotics could elevate ARG abundance. Also, operational enhancements, such as employing two-stage digestion, have shown promise in improving ARG removal. However, certain pretreatment methods, like thermal hydrolysis, may exhibit a rebounding effect on ARG levels. Overall, this review systematically addresses current challenges and offers future perspectives associated with the fate of ARGs in AD systems.

**Graphical Abstract:**

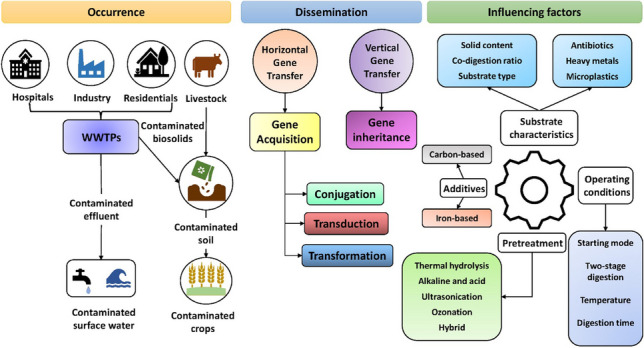

## Introduction

The increase in the abundance of antibiotic resistance genes (ARGs) and antibiotic resistant bacteria (ARBs) in both natural and engineered systems has raised serious human health concerns. Antimicrobial resistance (AMR) accounted for 1.2 million deaths worldwide in 2019 (Murray et al. 2022). The rise of superbugs harboring resistance against multiple drugs is especially concerning (Barancheshme and Munir 2019). Wastewater treatment plants (WWTPs) receive an influx of antibiotics from a plethora of sources ranging from municipal wastes, livestock wastes, hospitals, and the pharmaceutical manufacturing industry (Oberoi et al. 2019). The presence of these antimicrobial compounds exerts selective pressure on the microbes leading to the development of resistance against them (Van Hoek et al. 2011; Berglund 2015). Anthropogenic activities are the primary source of ARG distribution in the environment coupled with the limited capabilities of the wastewater treatment plants (WWTPs) in adequately removing these emerging contaminants. WWTPs have been considered the main reservoirs of ARGs and ARBs which can proliferate and spread in the environment (Devarajan et al. 2015; Di Cesare et al. 2016). Livestock industry wastes such as manure have been reported to be a hotspot for antimicrobials and ARGs, and they can be distributed in the ecosystem through land applications (Congilosi and Aga 2021). Recently, ARGs have also been detected in food waste (Cui et al. 2020). Additionally, there are many studies on the occurrence and detection of ARGs in the environment, including aquatic and terrestrial environments (Pruden et al. 2013; Su et al. 2017; Fang et al. 2017).

Various treatment methods have been investigated to remove ARGs in WWTPs. Disinfection techniques such as chlorination (Wang and Chen 2022) and UV light (Das et al. 2022) are common methods to eliminate ARBs in wastewater, but ARGs may survive even after disinfection (Huang et al. 2011). Also, advanced oxidation processes have gained attention due to their efficacies (Guo et al. 2017). In addition to chemical processes, different biological treatments have been utilized in order to treat ARGs. Anaerobic digestion (AD) is one of the biological treatment methods in which organic matters are degraded by microorganisms in the absence of oxygen to biogas and digestate. Recently, AD has gained more attention because of its bioenergy production potential (Zhang et al. 2015). In addition to bioenergy production, the digestate is also used as biofertilizer for agricultural purposes making the study of the fate of ARGs in AD processes more important (Rehman et al. 2018). AD processes including mono-digestion and co-digestion depend on several parameters such as substrate type, operating parameters, and presence of additives, to name but a few, which can affect the fate of ARGs in AD processes. It is noteworthy that ineffective treatment and disposal methods can exacerbate public health by disseminating ARGs in the environment. Hence, there is a need for a comprehensive overview of the effects of influencing factors on the fate of ARGs in all AD-based processes.

In recent years, many studies have focused on the fate of ARGs due to the global public health concern. Few review articles have also been published recently on the fate of ARGs in various biological processes such as composting and AD (Youngquist et al. 2016; Cui et al. 2020; Congilosi and Aga 2021; Haffiez et al. 2022). However, a comprehensive review critically discussing the effects of various influencing factors on AD processes that can affect the fate of ARGs is required to shed light on research gaps. In this respect, this review aimed to provide an overview of the occurrence and dissemination of ARGs in addition to the fate of ARGs in anaerobic mono and co-digestion processes. Eventually, current challenges are discussed, and the prospects for future research studies are proposed.

## Occurrence of ARGs in AD systems

**Fig. 1:**
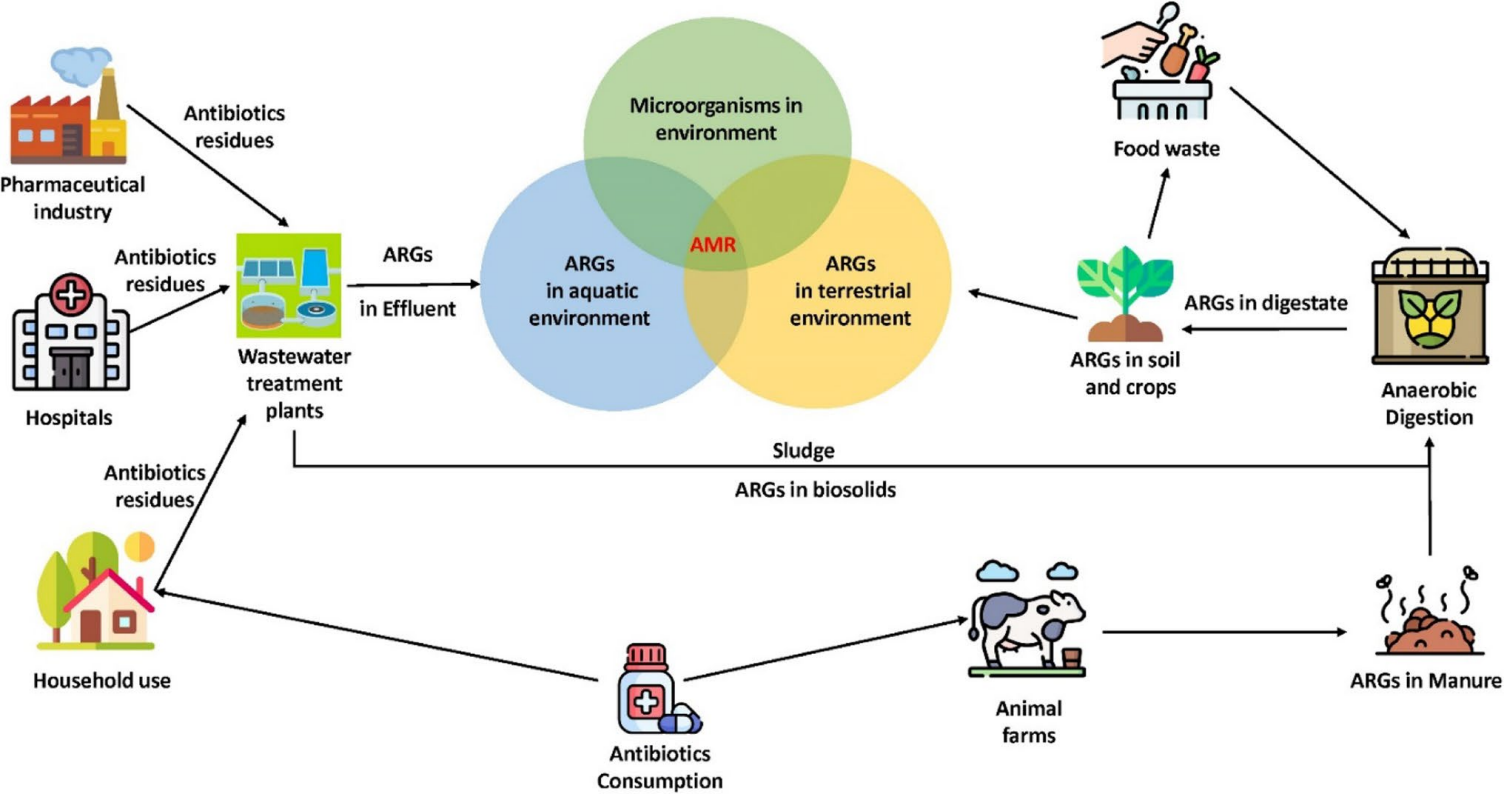
Occurrence of ARGs and AMR in the environment

Anthropogenic sources are the primary contributors to the occurrence of ARGs in the environment through the accumulation of residual antibiotics in wastewater systems from hospitals, households and livestock applications, and pharmaceutical manufacturing facilities (Barancheshme and Munir 2018). Figure 1 illustrates the main hotspots for AMR and ARGs in the environment. Since most WWTPs were conventionally not designed to treat emerging contaminants such as antibiotics and ARGs, a noticeable amount of antibiotic residues has been routinely detected in wastewater discharges. It creates optimal selection pressure on the microbes for the propagation of antimicrobial resistance and their further distribution through the system and in the environment results in the presence of ARGs in surface waters (Rodriguez-Mozaz et al. 2015). Selective pressure put by the residual antibiotics on the microbial community is one of the critical factors affecting the propagation and dissemination of ARGs in the environment. Still very little is known in terms of the in situ transfer mechanisms of ARGs in the natural environment and the influence of various abiotic and biotic stressors. WWTPs have become the reservoirs of ARGs across the spectrum (Table 1). Table 1 summarizes recently detected ARGs in various samples that are usually the feedstock of AD systems. According to the literature summarized in Table 1, tetracycline, beta-lactam, sulfonamide, and macrolide were the most studied antibiotics. Additionally, sulfonamides and tetracyclines are the most consumed antibiotics in the livestock industry which has led to the increasing detection of ARGs due to these routinely used antibiotics (Wu et al. 2020; Zhang et al. 2020b).

**Table 1:** Detected ARGs in different AD feedstocks

Sample type	Antibiotic class	Detected ARGs	Reference
Sewage sludge	Tetracycline, sulfonamide, macrolide, quinolone	*tetM*, *tetQ*, *tetA,tetB*, *tetE*, *tetG*, *tetH*, *tetX*, *sul1*, *sul2*, *emB*, *ermC*, *qnrD*	Lee et al. (2017)
	Tetracycline, sulfonamide, chloramphenicol, beta-lactam, vancomycin, aminoglycoside, MLSB (Macrolide-Lincosamide-Streptogramin B), multidrug	202 ARGs subtypes	Xu et al. (2020)
	β-Lactam, tetracycline, fluoroquinolone, sulfonamide	*bla*_*TEM*_, *bla*_*OXA*_, *bla*_*SHV*_, *tet*A, *tetM*, *qepA*, *aac(6′)-Ib-cr*, *sul1, sul2*	Hubeny et al. (2021)
Dairy manure	Tetracycline, sulfonamide	*tetO*, *tetW*, *sul1*, *sul2*	Wallace et al. (2018)
	β-Lactam, macrolide, sulfonamide, tetracycline	*bla*_*CTX-M-1*_, *ermB*, *sul1*, *tetA*, *tetW*, *tetX*	Mckinney et al. (2018)
Cattle manure	Sulfonamide, penicillin, β-lactam, macrolide, tetracycline	*sul1*, *sul2*, *ampC*, *bla*_*OXA-1*_, *bla*_*NDM1*_, *ermB*, *ermF*, *tetO*, *tetW*	Ge Lou et al. (2020)
	Macrolide, sulfonamide, tetracycline	*ermF*, *ermB*, *sul1*, *sul2*, *tetX*, *tetW*, *tetQ*, *tetM*, *tetK*, *tetG*, *tetC*	Gou et al. (2021)
Swine manure	Tetracycline, sulfonamide, trimethoprim, MLSB, β-lactam, quinoline, chloramphenicol, aminoglycoside, polymyxin	36 ARGs	Cao et al. (2020b)
	Tetracycline, β-lactam, sulfonamide, macrolide	18 ARGs	Cao et al. (2020a)
Poultry manure	Aminoglycoside, β-lactam, quinolone, sulfonamide, tetracycline, macrolide	*aac(6′)-Ib-cr*, *bla*_*CTX-M*_, *qnrD*, *oqxB*, *sul1*, *tetA*, *tetX*, *ereA*, *ermB*	Riaz et al. (2020)
	β-Lactam, quinolone, sulfonamide, tetracycline, macrolide	16 ARGs	Guo et al. (2020)
Food waste	Tetracycline, sulfonamide, macrolide, quinolone	14 ARGs	Lee et al. (2017)
	Multidrug, sulfonamide, tetracycline, aminoglycoside macrolide, β-lactam	*mexF*, *sul1*, *tetQ*, *aadA*, *strB*, *mefA*, *ermB*, *bla*_*CTX-M*_, *bla*_*TEM*_, *bla*_*OXA*_	Wang et al. (2020a)

Antibiotics are routinely used for clinical applications for humans and in the livestock industry. However, a major portion of antibiotics (10–70%) consumed by humans and livestock are excreted in unmetabolized forms which end up in the treatment systems (Selvam et al. 2012). However, with the limited antibiotic degradation efficacy of biological wastewater treatment facilities, they eventually end up in sludge management facilities and AD systems from WWTPs (Li and Zhang 2010). Antibiotics’ physico-chemical characteristics are the guiding factors influencing various adsorption mechanisms and their interaction with sludge particles (Oberoi et al 2019). Thus, biosolids, in addition to WWTP effluents, have turned into another main source of antibiotics and a route for ARG dissemination in the environment (Zhu et al. 2013).

## Mechanism of action and dissemination of ARGs

Antibiotics inhibit bacterial growth through various modes of action (Liwa and Jaka 2015). As shown in Table 2, antibiotics can inhibit protein synthesis such as aminoglycoside, interfere with cell wall synthesis like β-lactam, or inhibit DNA and RNA synthesis like quinolone. Over a period, bacteria evolved and adapted to gain resistance against these antibiotics using different mechanisms (Table 2). Generally, resistance is developed through chromosomal DNA mutation and is passed on to progeny via vertical gene transfer (VGT). Competent microbes in the environment can also take up free DNA released after cell lysis harboring ARGs to acquire resistance which gives them a selective advantage through a process known as via horizontal gene transfer (HGT) (Van Hoek et al. 2011).

**Table 2:** Primary mechanisms of action of antibiotics and related resistance mechanisms in microorganisms

Antibiotic type	Primary mechanism of action	Resistance mechanisms	Reference
Aminoglycoside	Inhibition of protein synthesis (30S ribosomal subunit)	Active efflux, decreased permeability, ribosome alteration, enzyme modification	Magnet et al. (2001) Taber et al. (1987) Poehlsgaard and Douthwaite (2005) Shaw et al. (1993)
β-Lactam	Interference with cell wall synthesis	β-Lactamase expression Altered penicillin-binding proteins (PBPs)	Van Hoek et al. (2011)
Chloramphenicol	Inhibition of protein synthesis (50S ribosomal subunit)	Enzymatic inactivation by acetylation Inactivation by phosphotransferases Permeability barriers Efflux pump Target site mutation	Murray and Shaw (1997) Schwarz et al. (2004)
Glycopeptide	Interference with cell wall synthesis	Modified peptidoglycan precursors resulting in low binding affinity	Van Hoek et al. (2011)
Macrolide-lincosamide-streptogramin B	Inhibition of protein synthesis (50S ribosomal subunit)	rRNA methylases Efflux pump Inactivating genes Including esterases, lyases, phosphorylases, transferases	Roberts et al. (1999) Roberts (2008)
Quinolone	Inhibit DNA synthesis	Decreased permeability, efflux pump, mutation of DNA gyrase and topoisomerase IV Plasmid-mediated efflux pump	Van Hoek et al. (2011) Martínez-Martínez et al. (1998)
Streptothricin	Inhibition of protein synthesis	Acetylation by acetyltransferases	Van Hoek et al. (2011)
Sulfonamide	Inhibition of intermediary metabolic pathways	Chromosomal mutation Plasmid-mediated genes	Sköld (2000) Swedberg and Sköld (1983)
Tetracycline	Inhibition of protein synthesis (30S ribosomal subunit)	Energy-dependent efflux pump Ribosomal protection proteins (RPPs) Enzymatic inactivation	Van Hoek et al. (2011)
Trimethoprim	Inhibition of intermediary metabolic pathways	Plasmid-mediated genes	Van Hoek et al. (2011)

**Fig. 2:**
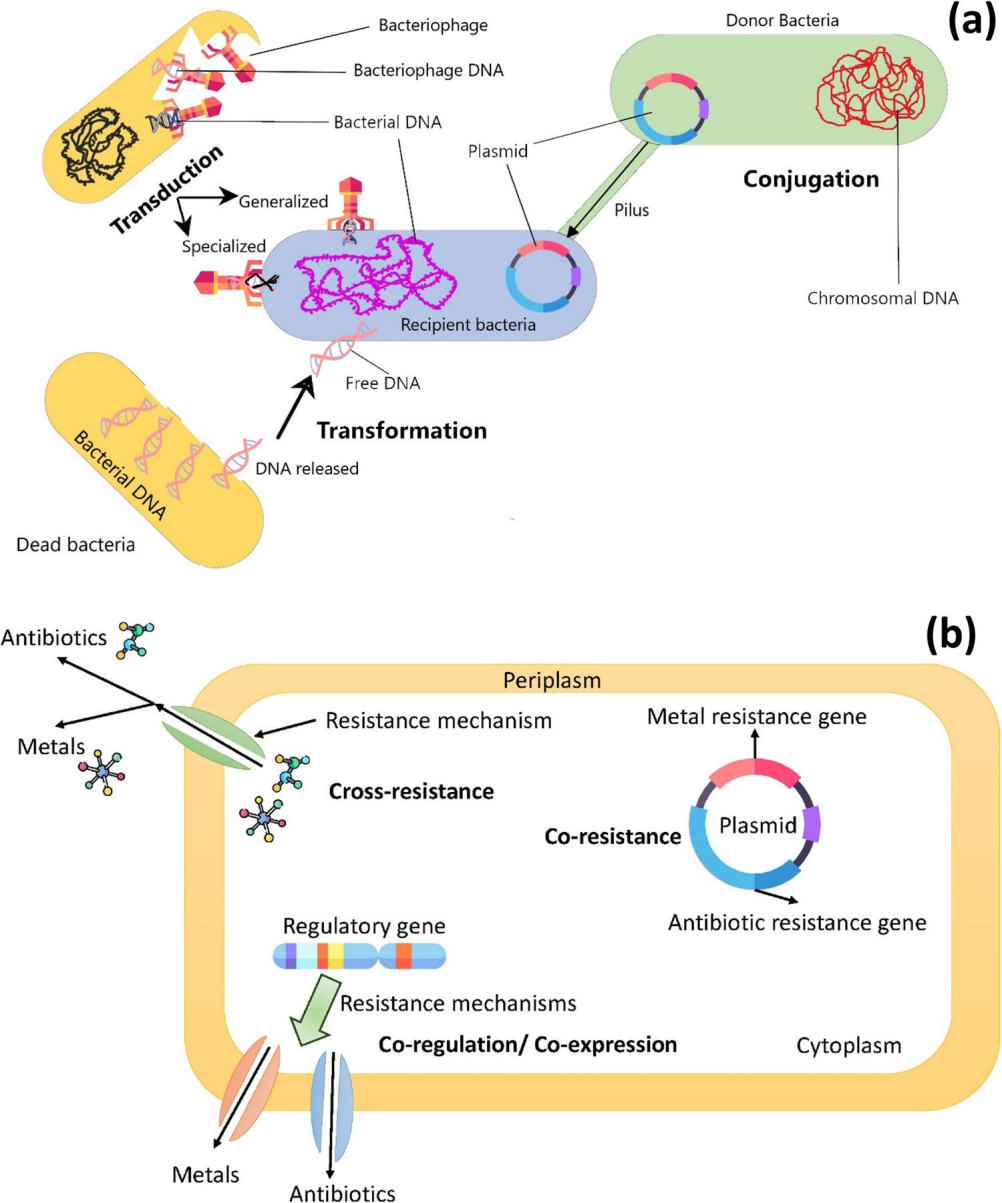
The mechanisms in **a** HGT of ARGs; **b** metal and antibiotic resistance co-selection

Transformation, transduction, and conjugation are the primary mechanisms for HGT (Barancheshme and Munir 2018). Figure 2a presents a schematic flow of HGT mechanism. Conjugation happens through cell-to-cell contact between the donor and the recipient through pilus (von Wintersdorff et al. 2016). Transduction is a process in which the DNA is transferred from the donor cell to the recipient cell through bacteriophage. Thus, bacteriophages play a key role in this mechanism (Emamalipour et al. 2020). Transduction can be generalized or specialized. In generalized transduction, any bacterial DNA (chromosomal or plasmid) can be packaged in a bacteriophage and transferred to another bacterium. Unlike generalized transduction, in specialized transduction, specific fragments of DNA are picked up by bacteriophage and transferred to the recipient bacterium (Chiang et al. 2019). During the transformation mechanism, bacteria uptake, integrate, and functionally express any freely available DNA in the environment (Thomas and Nielsen 2005). Mobile genetic elements (MGEs), including transposons, plasmid, and integron, can result in rapid dissemination of AMR through HGT. MGEs, especially class 1 integron (*intl*1), are usually considered a good indicator of HGT occurring in processes such as AD (Domingues et al. 2012).

Unlike antibiotics, metals have been available in the natural environment for billion years and resistance to metals and metalloids is a common phenomenon in many microorganisms (Pal et al. 2017). Interestingly, manifold studies have shown that environmental metal contamination results in the proliferation and expression of antibiotic resistance (Baker-Austin et al. 2006; Pal et al. 2017). Generally, co-selection of metal and antibiotic resistance in bacteria can occur via three mechanisms cross-resistance, co-resistance, and co-regulation (Fig. 2b). Cross-resistance can occur when one resistance mechanism such as efflux pump develops resistance to different compounds (metals and antibiotics) at the same time (Pal et al. 2015). In co-resistance, resistance genes (two or more) are located exactly on the same genetic element such as a plasmid, transposon, or integron (Chapman 2003). For example, *strB* (streptomycin resistance), *merE*, and *merD* (mercury resistance) are located on the pHCM1 plasmid which can confer metal and antibiotic resistance (Baker-Austin et al. 2006). Co-regulation (co-expression) is more complex than the other two mechanisms. It occurs when regulatory genes that are transcriptionally linked control multiple resistance genes to confer resistance to different compounds like metals and antibiotics (Pal et al. 2017). For instance, the linkage between *mex* and *czc* operons results in resistance to cobalt, zinc, and cadmium along with imipenem (Baker-Austin et al. 2006).

## Factors affecting the fate of ARGs in AD processes

**Fig. 3:**
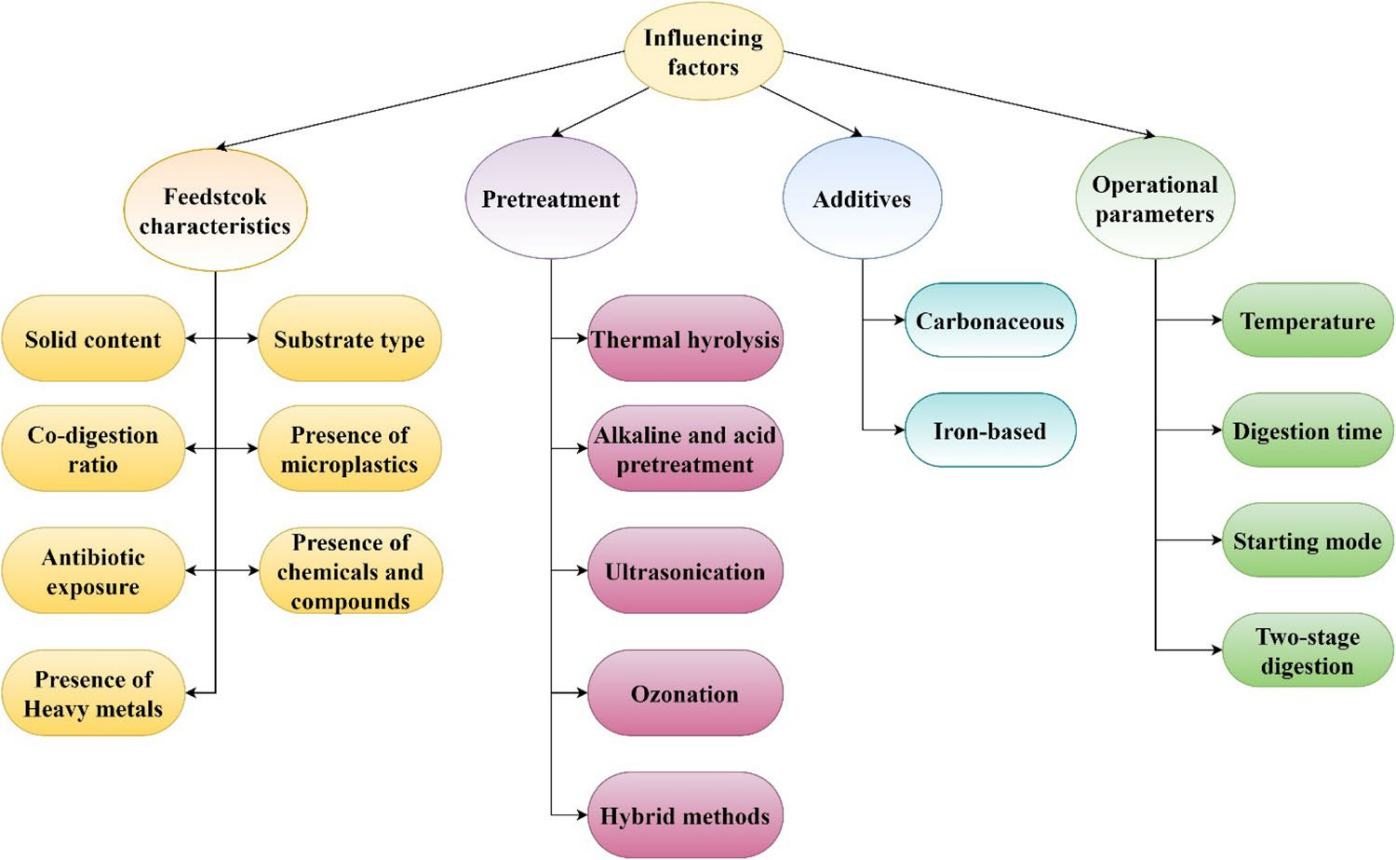
Influencing factors on ARGs fate during AD process

Microbial consortia are the main active components for an efficient AD process in addition to other abiotic environmental parameters. As ARGs are harbored by some of these microbes, any change in the community dynamics can affect the fate and removal of ARGs in the process. In this section, various factors influencing ARG fate in AD-based processes have been evaluated and are classified into four main groups: substrate characteristics, pretreatments, additives, and operating parameters. Figure 3 outlines these influencing factors on the fate of ARGs.

### Feedstock characteristics

AD feedstocks range from animal waste, agricultural waste, food waste, and sewage sludge among others. AD feedstocks have variable characteristics such as fat and oil content, ammonia concentration, or different solid contents which can affect the AD processes. Feedstock characteristic is one of the main factors affecting ARGs’ fate because it can influence the proliferation of microbial communities which can impact the fate of ARGs (Cui et al. 2020). In this section, the effects of such characteristics including solids content, co-digestion ratio, presence of antibiotics, and heavy metals on the fate of ARGs in AD processes are discussed.

#### Solid content

Solid content is one of the main factors affecting the AD process. Based on solid content, AD processes are classified into wet AD (solid content < 10%) and dry or solid-state AD (solid content > 20%). Solid-state AD (SSAD) was reported to enhance the performance of AD digesters and reduce the reactor volume in comparison to wet-AD (WAD) (Ziaee et al. 2021). Although the effect of solid content on AD performance has been well studied, its effects on the fate of ARGs are scarcely reported. Sui et al. (2018) investigated the fate of different ARGs (*bla*_TEM_, *ereA*, *ermB*, *ermF*, *mefA*, *sul1*, *sul2*, *tetG*, *tetM*, *tetA*, *tetX*, *mcr*-1, aac(6′)-Ib-cr) during AD of swine manure at different solid contents (4–14%). They reported a higher abundance of ARGs with increasing solids content. As the total solids (TS) increased, FA and volatile fatty acids (VFAs) also increased, which may have impacted the reduction of ARGs at higher solids load. Change in microbial community with increased dominance of acetate and propionate-producing bacteria, syntrophic acetate bacteria (SAOB), and hydrogenotrophic methanogens at high solids content. Additionally, as VFAs accumulated in the AD process, proteobacteria, the main carrier of *intI1* gene, increased in the system, particularly in the high solid feedstock. Mobile genetic carriers such as *intI1* gene are the key drivers for the acquisition of ARGs as observed at high solid AD process.

In contrast, Sun et al. (2019c) reported that SSAD (22% TS) outperformed WAD in the removal of *tetC*, *sul2*, *ermQ*, *ermX*, *qnrA*, and *aac(6′)-ib-cr* after AD of cattle manure. SSAD showed better removal of MGEs such as *intl1*, *intl2*, and ISCR1 while MGEs were enriched during LAD. This was attributed to the decrease in the abundance of Firmicutes and *Proteobacteria* as the main bacterial hosts for ARGs. Nonetheless, it is still unknown how higher solid content affects the microbial community and ARG removal in combination with other contributing factors which needs further investigation. In another study, Wang et al. (2021b) reported an initial enrichment of ARGs during dry AD (20% TS) of pig manure and food waste followed by improved removal rates towards the end as compared to WAD. The authors reported that the cellular protection mechanism in the dry AD increased whereas the efflux pump and antibiotic deactivation mechanisms decreased. Also, one theory for higher removal of ARGs in dry AD can be attributed to the fact that microbial mobility in dry AD systems is usually less than wet systems which can affect the HGT mechanism in the propagation of ARGs (Zhang et al. 2018a).

#### Co-digestion ratio

Co-digestion ratio is one of the critical parameters in the anaerobic co-digestion (AcoD) process. Different ratios can change the substrate characteristics such as microbial communities and, subsequently, ARG fate in the system (Jiang et al. 2022). Co-digestion has been used as one of the efficient methods to overcome the drawbacks of mono-digestion of substrates. Several researchers have also investigated the fate of ARGs in the co-digestion of different substrates (Zhang et al. 2018c; Wang et al. 2022c; Manyi-Loh et al. 2023). However, these studies primarily focused on AD performance during the co-digestion process rather than comparing the fate of ARGs. In the future, empirical studies are required to compare the impact of co-digestion with mono-digestion to provide a more comprehensive overview of the fate of ARGs in AD systems.

Few studies have investigated the effect of co-digestion ratios on ARGs’ profile. Jiang et al. (2022) studied various portions (2:1, 1:1, 1:2) of gentamicin mycelial residue to wheat straw on a VS basis. The results demonstrated that the relative abundance of total ARGs (gene copies/copies of 16S rRNA) did not increase as the wheat straw portion increased in the system. The same trend occurred for MGE fate in the system and MGE abundance declined by decreasing the mixing ratio. Additionally, 1:1 ratio was found to enhance methane and biogas production along with increased ARG and MGE removal and impeding conjugation process. Ma et al. (2021) investigated the different oil-to-FW ratios (0:1, 0.1:0.9, 0.3:0.7, 0.5:0.5 on VS basis) on the fate of ARGs. The results showed that lipid stress could improve the removal of total ARG abundance. Also, as the methane production proceeded to the final stage, the relative abundances of all ARGs including *intl1* reduced, especially at the lipid content of 30% (0.3:0.7 ratio) which suggests that lipid stress inhibits ARG proliferation at the final stage of the AD process. Community profile through network analysis demonstrated that *Firmicutes*, *Bacteroidetes*, *Synergistetes*, and *Proteobacteria* were the potential hosts for ARGs, and a decrease in their abundance was directly related to the reduction of ARGs in the process.

In another study, Song et al. (2017) investigated the effect of different mass ratios of swine manure to wheat straw (3:7 (C1), 5:5 (C2), and 7:3 (C3)) on the fate of ARGs. The results showed that the absolute abundance of total ARGs and *intl1* increased in this order: C3 > C1 > C2. Interestingly, C_3_ was not the optimum ratio for biogas production indicating that ARG removal should be simultaneously accounted for in future studies. Moreover, a higher removal of sulfonamide genes was reported compared to tetracycline and macrolide genes. Microbial community analysis also revealed that the main phyla in the process were *Firmicutes*, *Proteobacteria*, *Bacteroidetes*, *Actinobacteria*, and *Spirochaetes*.

#### Exposure to antibiotics and anti-infective agents

Routine detection of antibiotics and associated antimicrobial compounds coupled with the limited removal efficiency of conventional treatment methods have made them emerging contaminants of concern (Rosenfeld and Feng 2011). Antibiotics have been widely detected in animal waste and sewage sludge, which eventually end up in anaerobic digesters (Zhang and Li 2011; Wohde et al. 2016). The presence of antibiotics in these AD feedstocks exerts selective pressure on microorganisms leading to the proliferation of ARBs/ARGs in the system (Szczepanowski et al. 2009). The presence of antibiotics in the AD process is directly related to specific ARG presence and proliferation in AD systems. For instance, there are reports on the presence of protein synthesis interfering antibiotics such as oxytetracycline (Wang et al. 2016b; Tian et al. 2018), tetracycline (Aydin et al. 2015; Xiong et al. 2017), and erythromycin (Aydin et al. 2016) positively correlated to the increase in resistant genes such as *tetQ*, *tetW*, *tetM*, *tetO*, and *ermB* imparting against these antibiotics due to the selective pressure. Moreover, because of the complex nature of the AD process and different feedstocks, the effects of antibiotics’ presence on ARG fate become complex, and further studies are required to robustly investigate the functional microbial communities and their correlation with ARG persistence and expression.

In a recent study, Sun et al. (2023) studied the effect of enrofloxacin at 4 and 8 mg/L on the dissemination of ARGs during AD of cattle manure. They observed that among 10 detected genes, except for *tetC* and *tetX*, the abundances of other genes increased during digestion. The authors also reported that when ARGs (such as *sul1* and *aac(6′)-ib-cr*) have a significant correlation with class 1 integron (*intl1*), they might have a quicker response to enrofloxacin stress. Flores-Orozco et al. (2020) investigated the effect of ceftiofur (250 mg/L) on the fate of *cmy-2* during AD of dairy manure. They reported that although the exposure to the antibiotic imposed selective pressure, the process was able to remove the *cmy-2* gene by 55% and 33% for control and test reactors after digestion, respectively. Additionally, Zhu et al. (2021a) reported that the presence of lincomycin (1000 and 5000 mg/L) led to an increase of lincomycin resistance genes (*ermB* and *ermF*) after digestion. However, the relative abundance of *lnuB* was reduced during the AD process of sludge because *lnuB* shows resistance through the O-nucleotidylation of the hydroxyl group of lincomycin. In contrast, *ermB* and *ermF* show resistance through ribosomal protection. In another study, Bai et al. (2019) used six kinds of sulfonamides and tetracyclines (2 mg/L each) and reported that they enriched the absolute and relative abundance of their associated ARGs (*tetA*, *tetW*, *sul1*, and *sul2*) in the AD process of sludge. However, the abundance of *tetM* and *intI1* decreased in the process. Also, Ni et al. (2020) revealed that the esterase synthesis (*ereA* and *ereB*), methylation (*ermF* and *ermG*), and phosphorylation (*mphA*) were the main resistance mechanisms, while efflux pump (*mefA*) was the minor resistance mechanism during AD of waste activated sludge in the presence of roxithromycin with the concentration of 1000 µg/L. According to the results, the relative abundances of *ereA*, *ereB*, *ermF*, *ermG*, and *mphA* increased, whereas *ermC*, *ermX*, and *mefA* reduced. In addition to antibiotics, feed additives like arsanilic acid have been used in the poultry and swine industry as anti-infective agents. Sun et al. (2017) reported that the concentration of 650 mg/kg of arsanilic acid could increase absolute abundances of *tetC*, *sul2*, *sul1*, *ermB*, *gyrA*, *(6′)-Ib-cr* and *intI1* in AD of pig manure. In addition, they reported a co-occurrence between *gyrA*, *aac(6′)-Ib-cr*, *sul1*, *ermX*, *intI1*, and *arsC* genes. *Firmicutes* and *Proteobacteria* were the possible potential hosts for ARGs.

The take-home message from the literature studied is that the presence of antibiotics can promote selective stress on microbial communities in the AD process. Although these studies were conducted at higher antibiotic concentrations, more research is needed at environmental concentrations and implementing better treatment methods for the degradation of these compounds.

#### Presence of heavy metals

Heavy metals have been widely detected in WWTPs and animal farms (Mu et al. 2015; Kinuthia et al. 2020). The potential for co-resistance and selective stress by heavy metals can significantly affect ARG fate (Hu et al. 2017). In this regard, evaluating the effect of heavy metals on the fate of ARGs becomes essential. If the bioavailability of heavy metals does not change in AD, the selective pressure probably enhances ARG dissemination (Gupta et al. 2022). Depending on the metal type, ARGs may enrich or reduce during AD. The presence of zinc was reported to enrich relative abundances of *qnrS*, *sul1*, *sul2*, and *drfA7* (Yang et al. 2020b); absolute abundances of *ermX*, *sul1*, *sul2*, *tetX*, *tetM*, *tetG*, *(6’)-Ib-cr*, *ermF*, *ermB*, *drfA7*, and drfA1 (Zhang et al. 2017d); and absolute abundances of *sul1*, *sul2*, *drfA1*, and *drfA7* (Zhang et al. 2017c). Also, it was reported that HGT could be facilitated when zinc is present in the AD system due to the enrichment of *intI1* and *intI2* genes (Zhang et al. 2017c, d; Yang et al. 2020b).

In a recent study, Zhang et al. (2023) investigated the effects of zinc (125 and 1250 mg/L) on ARG profile in AD of swine manure. The presence of zinc, particularly at 125 mg/L, increased the abundance of ARGs and genotypes. Aminoglycoside and MLSB resistance genes were higher at 125 mg/L and sulfonamide resistance genes were higher at 1250 mg/L. Interestingly, Pang et al. (2022) reported that while zinc nanoparticles (5 mg/g TS) increased the diversity of ARGs in AD of cattle manure, it reduced the total abundance of ARGs by 6.6%. Additionally, microbial chemotaxis decreased by 17% indicating that ARG transmission through microbial adaptability decreased in the presence of zinc nanoparticles.

Wu et al. (2020) studied the effects of three types of copper salt (organic, inorganic, and mixed) (800 mg/kg dry weight) on the fate of ARGs during thermophilic AD of swine manure. The results showed that ARG abundance reduced in the presence of inorganic and mixed salts while increased in organic salt. Regarding *intI1*, a reduction was obtained in mixed Cu salts, while the inorganic and organic salts promoted the proliferation of *intI1*. Overall, organic copper showed the highest dissemination risk of ARGs in the environment. Microbial analysis indicated that Cu salts only changed the relative abundances of phyla or genera in the process rather than changing the predominant phyla or genera. In another study, Sun et al. (2017) reported that the concentration of 650 mg/kg of arsanilic acid could increase absolute abundances of *tetC*, *sul2*, *sul1*, *ermB*, *gyrA*, *(6′)-Ib-cr*, and *intI1* in AD of pig manure. In addition, they reported a co-occurrence between *gyrA*, *aac(6′)-Ib-cr*, *sul1*, *ermX*, *intI1*, and *arsC* genes. *Firmicutes* and *Proteobacteria* were the possible potential hosts for ARGs.

Most of these studies were mostly focused on zinc and copper resistance genes. Nonetheless, other heavy metals such as nickel, cadmium, mercury, and chromium should also be investigated since the resistance genes to these metals have been detected frequently (Yang et al. 2020a). Furthermore, more environmentally relevant concentrations of heavy metals should be studied in the future to assess the effects of heavy metals in AD substrates.

#### Substrate type

Since AD is one of the most common treatments for biosolids and biowastes, different substrates, including animal waste, food waste, yard waste, and sewage sludge, are digested in the system. Therefore, substrate type is another factor that should be taken into account when dealing with ARG fate. In this regard, Zhang et al. (2019c) studied the fate of 12 ARGs (*sul1*, *sul2*, *ermB*, *ermF*, *ereA*, *mefA*, *bla*_CTX-M_, *bla*_TEM_, *tetM*, *tetG*, *tetX*, and *mcr-1*) during AD of pig manure, chicken manure, and sewage sludge to assess the effect of substrate type on ARG fate. They reported that the relative abundance of total ARGs after digestion increased in pig manure (6.14 times) and chicken manure (2.65 times) while it reduced (8.95%) in sewage sludge. The AD process could remove *intI1*, indicating HGT was impeded after AD for all substrate types. The AD process was also efficient in the removal of heavy metal resistance genes (*merA*, *pcoA*, *arsC*, *czcA*), but the gene types varied between animal manure and sewage sludge. For instance, *pcoA* which confers resistance to copper dominated animal manure while *czcA* which confers resistance to Cd, Zn, and Co was dominant in sewage sludge. This difference can be attributed to the higher consumption of copper in the livestock industry.

The results demonstrated that substrate type could influence the initial load and eventual ARG fate during the AD process. However, the initial microbial communities in the substrate did not affect the ARG fate significantly while physicochemical parameters such as protein and cellulose contents were found to be more important. However, more studies are required to fully understand the variations as a function of various substrate types. In Table 3, the changes in ARGs during AD of different substrates are shown.

**Table 3:** The changes in ARGs during AD of different substrate types

Substrate type	AD operating conditions	ARGs increased	ARGs decreased	No change	Reference
Pig manure	Batch mesophilic AD for 44 days with a total TS of 8%	*tetM*, *tetX*, *mefA*, *ermF*, *sul2*	*tetG*, *ermB*, *bla*_*TEM*_, *mcr-1*, *intl1*	*sul1*	Zhang et al. (2019c)
Chicken manure		*tetM*, *tetX*, *mefA*, *ermF*	*tetG*, *ermB*, *bla*_*TEM*_, *mcr-1*, *sul2*, *intl1*	*sul1*	
Sewage sludge		*tetG*, *ermF*, *sul2*	*tetM*, *tetX*, *mefA*, *bla*_*TEM*_, *mcr-1*, *sul1*, *intl1*	*ermB*	
Cattle manure	Full-scale mesophilic AD digester With a digestion time of 20–30 days	*ermB*	*intl1*, *tetA*	*sul1*, *tetW*	Burch et al. (2022)
Cattle manure	Batch mesophilic AD for 64 days with total solids of 5%	*tetA*, *tetB*, *tetG*, *tetO*, *tetQ*	*tetM*, *tetW*	–	Agga et al. (2020)
Poultry litter		*tetM*, *tetW*, *tetO*	*tetQ*	*tetA*	
Swine manure		*tetA*, *tetG*, *tetM*	*tetO*, *tetW*	*tetQ*	
Cattle manure	Full-scale mesophilic AD digester with 22 days retention time	–	*sul1*, *sul2*	*tetO*, *tetW*	Wallace et al. (2018)

#### Presence of microplastics

Microplastics (MPs) have become an emerging contaminant of concern for environmental pollution, and they have also been detected in biosolids and biowastes (Sun et al. 2019b). Thus, their presence in AD process is inevitable. However, the knowledge governing the fundamental mechanisms affecting the fate of ARGs in AD systems in the presence of MPs is limited. Since antibiotics have been reported to adhere to the surface of MPs, one hypothesis is that these antibiotics can promote selective stress and ARG expression (Yu et al. 2020). Bacterial biofilm attachment on MPs has been found to facilitate ARGs spread through HGT (Zhang et al. 2020c). Additionally, studies on the effect of MPs on microbial communities revealed that MPs could increase bacterial abundance which might be another factor for ARG enrichments (Shi et al. 2020). However, most studies were conducted on spherical polyethylene microplastics (PE-MPs) while other shapes and materials are also detected in the environment.

Wang et al. (2023) showed that spherical PE-MPs with the size of 40–48 μm enriched total ARG abundance at different concentrations (50, 100, and 200 particles/g-TS) after thermophilic AD of SS. MGEs were also observed to increase 67.5% under MPs’ presence. In another study, Luo et al. (2023) also observed that PE- and polyvinyl chloride (PVC) MPs (200 μm) at 30 particles/g TS could increase ARG abundance in AD of SS by 14.8% and 23.6%, respectively. Also, the results demonstrated that PVC-MPs enriched human pathogen bacteria (*A. baumannii*) and methanotrophs more than PE-MPs. These results indicate that the pool of MPs investigated should be expanded in the future to understand the effects of MPs on ARG transmission.

Shi et al. (2022) investigated the effects of spherical polyethylene MPs of 180 μm and 1 mm at 100 mg/g TS on AD of waste-activated sludge. The presence of MPs, especially 1 mm, enriched bacterial communities by increasing the abundance of hydrolytic bacteria and acidogens. Except for *tetQ*, the abundances of *sul2*, *tetO*, *tetW*, and *bla*_OXA_ decreased after digestion. However, the presence of MPs increased the abundance of ARGs compared with control which can be attributed to the enrichment of ARG hosts in the system. In another study, Zhang et al. (2021b) studied the effect of spherical polyethylene MPs (diameter < 400 µm) by adding 1 g/L MPs to the AD system of dairy waste at thermophilic (55 °C) and hyperthermophilic (65 °C) conditions. The results indicated that the presence of MPs in the AD system hindered the removal of ARGs and increased their proliferation throughout the process. The authors also found that *intI1* increased in the system indicating promotion of HGT. Besides, the reduction of efflux pump-associated genes like *tetC* and *tetG* and ribosomal protection protein genes such as *tetW* was hindered by MPs. The possible reasons for this phenomenon are the antibiotics that can adhere to MPs’ surface, increasing the selective pressure on resistant microbes, causing enrichment and shifting in microbial community on the surface of MPs. MPs increased the abundances of *Bacteroidetes* and *Spirochaetes* but decreased *Firmicutes* and *Proteobacteria* abundance under thermophilic conditions. This effect can be attributed to the release of reactive oxygen species or bisphenol-A during AD (Chen et al. 2023). In contrast, under hyperthermophilic conditions, the abundance of the bacterial communities did not change dramatically. Additionally, MPs shifted the potential hosts for ARGs. The hosts of *tetC* changed from *Tepidimicrobium* and *Symbiobacterium* to *Cladiocoprobacter* when MPs were present in the system. Also, the potential host for *tetG* disappeared in the presence of MPs. The probable reason for this change in the microbial community was attributed to the accumulation of ammonia because of MPs.

#### Presence of ammonia and artificial sweeteners

In addition to the above section, some other compounds have been found to affect the fate of ARGs but only a few studies have discussed these compounds. Ammonia, especially free ammonia (FA), has been reported to be a critical parameter in AD processes influencing its stability and efficiency (Yang et al. 2019; Ziaee et al. 2021). Regarding ARG fate, ammonia stress was reported to have a positive effect on the reduction of some ARGs in the AD process since FA can inhibit antibiotic efflux by reducing membrane permeability (De Vrieze et al. 2015). Since FA limits the major facilitator superfamily (MFS) efflux pump through changing proton gradient, ARGs related to extracellular transport mechanism will be impacted in AD processes under ammonia stress (Chen et al. 2014). On the other hand, FA can enhance enzymatic modification in microbes resulting in the enrichment of antibiotic target alteration that can give rise to antimicrobial resistance such as the Erm 23S rRNA methyltransferase gene family (Jia et al. 2017; Zhang et al. 2020a). In a study, Zhang et al. (2020a) observed that macrolide-lincosamide-streptogramin B (MLSb) genes (*ermB*) enriched while tetracycline genes (*tetL*) were reduced in AD of swine manure under ammonia stress because *tetL* belongs to MFS efflux pump that was limited under ammonia stress (1500–3000 mg/L), but *ermB* belongs to Erm 23S rRNA methyltransferase which was facilitated by the presence of ammonia. Additionally, ammonia stress was reported to have a limited effect on aminoglycoside ARGs. The authors reported that although the control process could decrease MGEs, the presence of ammonia increased conjugative plasmids in test reactors. On the other hand, pKKS825 plasmids were reduced during ammonia stress. This plasmid is associated with antibiotic efflux and carries resistance genes like *tetL*, *dfrK*, *vagC*, and *aadD*. Thus, the reduction of these ARGs was related to the reduction of the plasmid. Concerning HMRGs, the ones associated with the efflux system, such as copper resistance genes, reduced under ammonia stress as reported by the authors.

Due to the widespread use of artificial sweeteners as sugar substitutes, they have been detected in sewage sludge and animal manure frequently (Buerge et al. 2011; Luo et al. 2019). Yang et al. (2023) found that acesulfame at different concentrations (1, 10, and 100 mg/L) could enrich the abundances of ARGs and MGEs, and the number of potential hosts during AD of synthetic wastewater. The main reason was attributed to the DNA damage because of ROS (reactive oxygen species) production which activates the DNA damage response (SOS response) system in microbes. Additionally, the authors reported that acesulfame contributed to increased cell membrane permeability in the system which can accelerate HGT in the system. Although there are various types of artificial sweeteners in the market, only one study has covered their effects on ARGs in AD processes. Thus, future studies on other kinds such as sucralose, aspartame, and saccharin are needed.

### Effect of sludge pretreatments on ARGs

AD processes can degrade organic matter to produce biogas and less harmful digestate. However, the efficiency is hampered by the poor biodegradability of the substrates (Zhang et al. 2021c). Adequate pretreatment techniques for substrate such as thermal hydrolysis, ultrasonic wave, ozone, and pH adjustment have been used to improve the waste’s biodegradation and hydrolysis to enhance biogas production (Ariunbaatar et al. 2014; Lee et al. 2019; Villamil et al. 2020). Several studies have also investigated ARG fate after these pretreatment methods which can be classified into five groups: thermal hydrolysis, ultrasonication, ozonation, alkaline, and hybrid pretreatment. Table 4 summarizes details of different pretreatment methods.

**Table 4:** ARGs fate in anaerobic digestion processes with pretreatment

Sample type	Pretreatment method	Operational conditions	Target genes	ARG dynamics	Reference
Sewage sludge	Thermal hydrolysis	Hydrolyzed at 160 °C and 0.6 MPa for 30 min/digested in mesophilic and thermophilic condition	283 ARGs	Pretreatment significantly decreased the abundance of genes (> 94%, but after AD it increased slightly	Sun et al. (2019a)
Waste activated sludge	Alkaline	Treated with 0.04 g NaOH/g.TS at room temperature for 24 h Digestion time = 30 days	*tetA*, *tetC*, *tetB*, *tetG*, *tetM*, *tetO*, *tetQ*, *tetS*, *sul1*, *sul2*, *intl1*	Removal of total ARGs absolute abundance increased by 15.6% Total ARGs relative abundance decreased by 49.8%	Wang et al. (2019a)
Secondary sludge	Free ammonia	Treated with 420 mg NH_3_-N/L at pH = 10 at room temperature for 24 h Digestion time = 45 days	*aac(6′)-Ib-cr*, *bla*_*TEM*_, *sul1*, *sul2*, *tetA*, *tetB*, *tetX*, *tetG*, *tetM*	Removal of *aac(6′)-Ib-cr*, *bla*_*TEM*_, *sul2*, *tetA*, *tetB*, and *tetX* increased by 17%, 58%, 19%, 52%, 42%, and 74%, respectively	Zhang et al. (2021c)
Thickened waste activated sludge	Free ammonia	Treated with 560 mgNH_3_/L at pH = 9.5 for 24 h at room temperature	*aac(6′)-Ib-cr*, *bla*_*TEM*_, *sul1*, *tetA*, *tetX*	The removal of targeted ARGs increased by 34–86%	Liu et al. (2023)
Swine manure	Free nitrous acid	Treated with nitrite (250 mg/L) at pH = 5 at 20 °C for 24 h Digestion time = 60 days	*tetA*, *tetG*, *tetO*, *tetW*, *tetX*, *qnrS*, *qnrA*, *sul1*, *sul2*, *intl1*, *intl2*	Total residual ARGs reduced to 1 log lower than control Tetracycline resistance genes removed by 75%	Liu et al. (2022)
Sewage sludge	Microwave	600 W at 50 rpm/heated from 20 to 100 °C/digested in mesophilic condition	*ampC*, *bla*_*CTX-M*_, *bla*_*SHV*_, *intl1*, *tetX*, *tetO*, *tetM*, *tetC*, *tetA*	Total absolute concentration decreased by 1.2%	Tong et al. (2016)
Dairy wastewater	Ozonation	With concentration of 4.2 mg O_3_/L at a flow rate of 600 ml/min/digested in mesophilic condition	*sulI*, *sulII*, *tetG*, *tetO*, *tetW*, *intl1*	The relative abundance of total ARGs decreased after all pretreatments, but absolute abundance increased	Chen et al. (2021)
	Ultrasonication (US)	At 200 W with a frequency of 40 kHz/digested in mesophilic condition			
	Ozone/US	Ozone concentration of 4.2 mg O_3_/L at 200-W ultrasound/digested in mesophilic condition			
Sewage sludge	Thermal hydrolysis	At 120 °C for 60 min in an autoclave/digested in mesophilic condition	*tetA*, *tetB*, *tetC*, *tetG*, *tetM*, *tetO*, *tetQ*, *tetS*, *sulI*, *sulII*, *intI1*	52.5% reduction in absolute abundance/52.57% reduction in relative abundance	Wang et al. (2019a)
	Alkaline	24 h at room temperature with alkaline solution of 0.04 g NaOH/g-TS/ digested in mesophilic condition		66.38% reduction in absolute abundance/49.99 reduction in relative abundance	
	Ultrasonication	At a power density of 0.15 W/ml for 30 min/digested in mesophilic condition		75.07% reduction in absolute abundance/59.80% reduction in relative abundance	
Pharmaceutical waste sludge	Ozonation	Ozone concentration of 9 mg/L at a flow rate of 2 L/min followed by AD for 15 days	*tetA*, *tetG*, *tetQ*, *tetW*, *tetX*, *intI1*	All reduced (0.5–1.5 log units) except *tetQ* and *tetW* which rebounded after AD	Pei et al. (2016)
	Thermal hydrolysis	At 170 °C and 8 bars for 30 min followed by AD for 15 days		All reduced by 0.5–3 log units	
Municipal waste sludge	Ozonation	Ozone concentration of 9 mg/L at a flow rate of 2 L/min followed by AD for 15 days		All reduced by 1–2.5 log units	
	Thermal hydrolysis	At 170 °C and 8 bars for 30 min followed by AD for 15 days		All reduced by 1–3 log units expect for *tetW*	
Waste activated sludge	Microwave-H_2_O_2_	At 600 W with H_2_O_2_ dose of 0.2 g/g TS/digested in mesophilic one- and two-stage AD	*bla*_*OXA-1*_, *bla*_*TEM*_, *ereA*, *ermB*, *ermF*, *mefA/E. sulI*, *sulII*, *tetG*, *tetM*, *tetX*, *intI1*	Pretreatment itself reduced absolute gene copies while increasing all genes' relative abundance After AD, absolute and relative abundance increased, but two-stage AD performed better	Zhang et al. (2017b)
Chicken manure	Microwave-activated carbon	At 1000 W with agitation rate of 50 rpm/digested in mesophilic condition	*tetA*, *tetB*, *tetM*, *tetO*, *tetQ*, *tetW*, *tetX*, *sul I*, *sul II*, *floR*, *cmlA*, *intI1*	87–95% of ARGs removed	Zhang et al. (2019f)
Sewage sludge	Alkaline	Treated with 6 M NaOH at pH = 10 for 1 h	*sul1*, *sul2*, *tetA*, *tetO*, *tetX*, *bla*_*TEM*_, *bla*_*SHV*_, *intl1*	Total ARGs achieved 1.0 log reduction	Zou et al. (2023)
	Thermal hydrolysis	Treated at 160 °C for 30 min		Total ARGs achieved 0.7 log reduction	
	Enzymatic pretreatment	Treated with α-amylase and protease at 10 g/g TS at 37 °C for 6 h		Total ARGs achieved 0.6 log reduction	

#### Thermal hydrolysis

Thermal pretreatments have been broadly applied to improve dewaterability and enhance the hydrolysis of the substrate in AD processes (Carrère et al. 2010; Cesaro and Belgiorno 2014). Thermal hydrolysis causes cell wall destruction and release of organic matter, enhancing substrate availability for microorganisms (Xue et al. 2015). Pathogen removal and heavy metal immobilization through complex anions have been achieved by thermal pretreatment (Wang et al. 2019b). It is also effective for wastes rich in carbohydrates and proteins (Barber 2016). The studied temperature ranges were 60 to 250 °C, while most studies reported optimum ranges of 150 to 180 °C (Hii et al. 2014).

The primary mechanism for removing ARGs after thermal hydrolysis was attributed to the hydrolytic destruction of DNA and microbial biomass due to high temperature and pressure in the pretreatment (Pei et al. 2016, Wang et al. 2019a). Moreover, thermal treatment of feedstock before running AD was beneficial for removing inhibitory compounds like antibiotics (Hassani et al. 2008; Zhang and Li 2018). Sun et al. (2019b) reported that thermal hydrolysis could reduce tetracyclines, macrolides, and lincosamides. However, quinolones were found to be more stable than other antibiotics. Most recently, Azizi et al. (2022) investigated the effect of thermal hydrolysis (80 and 160 °C) on ARG propagation in AD of SS exposed to different levels of polystyrene nanoplastics (50–150 µg/L). They reported that although nanoplastics enriched ARG abundances more than control, thermal hydrolysis (particularly at high temperatures) could decrease their abundances. A decrease in selective pressure from antibiotic residues can reduce the pressure on microbes to acquire resistance.

Literature reports demonstrated that most ARGs and MGEs were reduced after the pretreatment. However, their abundances increased again after the AD process which is called the rebound effect (Ma et al. 2011; Pei et al. 2016; Tong et al. 2018, 2019; Wang et al. 2019a; Sun et al. 2019a). Since the treatment significantly removes ARGs in the feedstock, the increase of resistance genes during AD could be attributed to the seed inoculum used in the systems (Ma et al. 2011; Pei et al. 2016). In this regard, tracking the microbial community structure evolution is a crucial factor affecting the fate of ARGs.

In addition to substrate degradation, thermal hydrolysis directly affects the microbial community. Ma et al. (2011) reported that *Firmicutes* became the dominant phylum after the thermal pretreatment, indicating that fermentation was facilitated during thermal hydrolysis. Also, regardless of the rebound effect, ARG abundance was still lower than in the untreated substrate. One possible reason for this decline is the reduced bacterial diversity so it could narrow down the potential host ranges of ARGs. Other studies also reported that *Firmicutes* were enriched after thermal pretreatment especially the class of *Clostridia* which was found to be correlated with *tetM*, *tetQ*, and *mefA/E* (Wang et al. 2019a; Tong et al. 2019).

#### Alkaline and acid pretreatment

Alkaline pretreatment includes treating substrate with an alkaline solution for a certain amount of time and at room temperature. Wang et al. (2019a) reported that alkaline pretreatment (24 h at room temperature with 0.04 g NaOH/g-TS) itself had a limited effect on the absolute abundance of ARGs before AD. On the contrary, the ARG removal in the pretreated sample with the alkaline method increased by 16% after AD. The possible reason for the low removal of ARGs by alkaline treatment can be attributed to the stability of DNA structure under high pH conditions (Williams et al. 2001). It is noteworthy that although alkaline pretreatment can kill bacterial cells, it cannot destroy DNA at pH levels lower than 12, probably because phosphate groups of DNA have repulsive interaction with the hydroxide at pH ≤ 12 (Magnusson and Frey 2000; Burch et al. 2017).

In addition to alkaline pretreatment with NaOH, free ammonia (FA) treatment has recently been applied to improve anaerobic digestion performance. The main factors that make FA pretreatment appealing for ARG removal are its cell permeability, killing ARBs and damaging DNA. Recently, Liu et al. (2023) reported that FA pretreatment of waste-activated sludge (560 mgNH_3_/L at pH = 9.5 for 24 h) followed by AD resulted in lower levels of pathogens and ARGs. After digestion of pretreated sludge, the removal extents of ARGs were *aac(6′)-Ib-cr* (28–44%), *sul1* (95%), *tetX* (94–95%), and *bla*_*TEM*_ (12–13%) with insignificant difference between treated and untreated sludge for *tetA*. The potential reasons for ARG removal were attributed to the ability of FA to cause damage to cell-free ARGs. Additionally, high pH and TAN concentration could cause oxidation stress resulting in lysis of ARGs host. In another study, Zhang et al. (2021c) also reported that FA pretreatment of sludge (with 420 mgNH_3_-N/L at pH = 10 for 24 h) could increase the removal of *aac(6′)-Ib-cr*, *bla*_*TEM*_, *sul2*, *tetA*, *tetB,* and *tetX* from sludge by 17–74%. However, *intl1* removal was not affected by the FA pretreatment. Overall, after digestion, the total absolute abundance of targeted ARGs declined by 40% in treated sludge of which 15% was due to FA pretreatment.

Free nitrous acid (FNA) is also another novel pretreatment method that has recently been investigated to improve the efficiency of AD systems. Liu et al. (2022) reported that FNA pretreatment of swine manure (250 mg NO_2_-N/L at pH = 5 for 24 h) at ambient temperature (20 °C) followed by AD resulted in an enhanced reduction of ARBs (three logs for tetracycline resistant bacteria), ARGs (75% for tetracycline resistance genes), and antibiotics (80% for tetracycline) after AD compared to untreated manure. The reduction was mainly attributed to cell lysis and macromolecular compound breakdown. However, direct removal of ARGs and antibiotics by FNA pretreatment was found to be the main reason for the removal of ARGs and it was not directly related to ARB reduction due to conformation from community structure analysis.

#### Ultrasonication

Ultrasonic pretreatment has gained attention in recent years. As a physical treatment, ultrasonic systems at frequencies 20–100 kHz produce cavitation bubbles at high-power densities. The collapse of bubbles results in high local temperature (up to 4700 °C) and pressure (up to 180 MPa) (Mischopoulou et al. 2016). Additionally, free radicals can be generated as the result of bubble collapse. In this respect, two mechanisms were proposed to be responsible for the destruction of microbial cells and organic matters: (i) pyrolysis of recalcitrant organic compounds; and (ii) oxidation via free radicals such as hydroxyl radicals (Abbasi and Razzaghi-Asl 2008).

The effect of ultrasonic pretreatment showed variable results on ARG fate. One study found no effect on ARG abundance before the AD process possibly due to the low power density that could not efficiently destroy EPS and cell walls upon ultrasonic application (Zhang et al. 2017a). Chen et al. (2021) reported that relative abundances of total ARGs after AD of dairy wastewater treated with ultrasonic decreased by 16%. However, absolute abundance of total ARGs increased by 0.06–0.71 log after AD of the pretreated samples. The possible reason for the decline in relative abundance was attributed to the decrease of the 16S rRNA genes, which is an indicator of bacterial abundance. In another study, Wang et al. (2019a) revealed that ultrasonic pretreatment decreased RA and AA of ARGs after AD of sewage sludge and outperformed thermal hydrolysis based on ARG reduction. Based on the reported results in the literature, there is still a knowledge gap about the possible reasons for the increase or decrease of ARGs after ultrasonic treatment. It is noteworthy that both studies reported that *intI1* reduced after AD, indicating HGT can be potentially reduced after pretreatment.

#### Ozonation

Ozonation is one of the conventional technologies that has been utilized in the treatment of various substrates such as pharmaceutical and personal care products (Carballa et al. 2007), and polycyclic aromatic hydrocarbons (PAHs) (Bernal-Martinez et al. 2009). Ozonation is a chemical treatment in which free radicals are produced and react with organic matter to degrade them (Silvestre et al. 2015). Also, ozone has been used broadly to disinfect water, wastewater, and sewage sludge (Oh et al. 2014; Zhuang et al. 2015). For instance, Oh et al. (2014) reported that 90% of ARBs were eliminated in synthetic wastewater using 3 mg/L of ozone. It should be noted that ozone attacks the unsaturated carbon bonds and amino acids in peptidoglycan, lipids, and proteins changing the microbial cell’s permeability (Dodd 2012). Also, it was reported that ozone could affect the hydrogen bonds of extracellular substances in sludge (Meng et al. 2018; Wang et al. 2020b). Therefore, ozone, especially at high concentrations, can penetrate ARBs and deactivate the nucleic acid (Zhao et al. 2020).

Pei et al. (2016) reported ozone pretreatment (0.1 g O_3_/g TS) combined with AD could remove more ARGs in pharmaceutical and municipal waste sludge (Table 4) compared with single AD or only ozonation treatments. The non-selective oxidation caused by ozone was considered the main possible reason for the reduction of targeted genes because ozone could first react with the cell envelope and after ARB removal could reduce ARG abundances. In this regard, the ozone concentration becomes a key factor since it increases the chance of ozone reaction with cytoplasm and cell envelope. Although subsequent AD process could remove more ARGs, *tetW* and *tetQ* rebounded after AD process which was mainly attributed to microbial community change due to chemical pretreatment.

In contrast, Chen et al. (2021) revealed that absolute abundances of total ARGs, particularly, *sul1* (0.34 log) and *tetW* (0.39 log), increased after AD of samples pretreated by ozonation (4.2 mg O_3_/L) (Table 4). Also, these results showed that the pretreatment could destroy bacterial cells and change bacterial community structure which could directly contribute to increased relative abundances of targeted ARGs. However, the relative abundance of total ARGs increased by only 5.53% after AD of pretreated samples while it increased by 112% in untreated samples. Additionally, the relative abundance of *intl1* did not change significantly after AD in ozone-treated samples. In this regard, further studies are required to shed light on the removal of ARGs in different feedstocks pretreated by ozonation.

#### Hybrid pretreatments

In addition to the methods mentioned, several studies have been conducted to assess the effect of a combination of pretreatments on ARG fate and removal in AD systems. In a recent study, Li et al. (2023) combined alkali-ultrasonication by first treating sludge samples with 0.04 g NaOH/g TS for 30 min followed by ultrasonication at 0.2 W/mL for 30 min. The results showed that the hybrid system could achieve over 85% bacterial population removal efficiency, in particular ARBs. The subsequent AD process further reduced the abundance of ARBs ranging from 36 to 83%.

Shin et al. (2022) reported that recuperative thickening (thickening a portion of the digestate and recirculating it to the digester) extends SRT which improves the removal of ARGs after digestion (64.3% removal of relative abundance). On the other hand, the combination of recuperative thickening with thermal hydrolysis (at 121 °C and 1.3 MPa for 15 min) reduces the removal of the relative abundance of ARGs (47.4%) compared to untreated SS samples (57.3%) after digestion.

Song et al. (2020) applied alkaline-thermal pretreatment on spectinomycin mycelial residues (SMRs) in an AD system. They reported that at temperatures above 90 °C and pH > 12, the abundance of ARGs and MGEs reduced in AD tests while temperatures below 90 °C and pH < 12 were ineffective in destroying ARGs in AD systems compared to untreated samples. They also reported that TS content is a crucial factor since TS can impede the diffusion of hydroxide in the mixture and, as a result, increases the pH level of the mixture to above 12. High alkalinity can hydrolyze the phosphodiester bond of DNA, resulting in the removal of ARG abundance.

Zhao et al. (2020) coupled ultrasound and ozonation treatment to investigate the fate of ARGs after AD of sludge samples contaminated by levofloxacin. They reported that the active radicals generated by the hybrid system were able to reduce ARBs and degrade levofloxacin in the substrate which eventually resulted in the reduction of *qnrA* and *qnrS* genes by 1–2 orders of magnitude in the following AD system. Furthermore, free radicals such as OH^·^ degraded nutrients that made microorganisms compete. Thus, the pathogenic genus level decreased after the pretreatment.

Microwave pretreatment has been demonstrated to be effective in cell destruction and VS removal in AD (Kuglarz et al. 2013; Liu et al. 2015). Zhang et al. (2017b) reported that although AA decreased, and RA increased after microwave-H_2_O_2_ pretreatment, AA and RA of ARGs enriched after AD of pretreated waste activated sludge. On the other hand, Zhang et al. (2019f) studied the pretreatment of chicken manure by a microwave-activated carbon system. They reported that the pretreatment system significantly improved the removal of the relative abundance of *tetB*, *tetM*, *tetO*, *tetQ*, *tetW*, and *tetX* (except for *tetA*) in AD of chicken manure. The main role of microwave in the pretreatment was attributed to the deactivation of host bacteria, while activated carbon had three roles. First, activated carbon facilitated the proliferation of methanogens rather than ARBs. Second, because of surface sites, bacteria aggregated, resulting in less mobile microorganisms but enhanced microbial activity. Finally, activated carbon has been reported to decrease the mobility of microbial communities due to the adsorption of bacteria which can impede the spread of ARGs in bacteria via HGT.

Recently, Zou et al. (2023) conducted alkaline, thermal, and enzymatic pretreatment on SS to investigate the fate of extracellular ARGs (eARGs) and intracellular (iARGs) (Table 1). In this comparative study, they reported that pretreatment-AD processes do not show a significant advantage compared with AD only. Except for intracellular *tetO*, the absolute abundances of other iARGs and eARGs decreased in alkaline-AD, enzymatic-AD, and single AD. Additionally, eARGs were found to be removed more than iARGs since iARGs reside in cells and nuclease access to them was limited. Moreover, the change in bacterial communities resulted in the proliferation of ARG hosts in AD systems which resulted in the reproduction of iARGs during AD.

### Effect of additives

The limitations in the AD process encouraged researchers to investigate different ways to enhance biogas production. One way to improve the process is the addition of organic or inorganic additives. Organic additives consist of biochar, microbial cultures, and enzymes, while inorganic additives include macro-nutrients, micro-nutrients, and carbon-based materials (Baniamerian et al. 2019). In addition to enhancing AD performance, up to date, several studies have investigated ARG fate during the AD process when additives were utilized. Typically, the studied additives can be divided into carbonaceous and iron-based materials. The results are presented in Table 5.

**Table 5:** Effect of different additives on ARGs fate in anaerobic digestion processes

Additive type	Additive dosage	Sample type	Operational condition	Targeted genes	ARG fate	Reference
Activated carbon	15 g/L	Chicken manure and food waste	Mesophilic semi-continuous CoAD for 30 days	*sul1*, *sul2*, *tetA*, *tetW*, *tetO*, *tetX*, *tetB*, *tetM*, *tetQ*, *cmlA*, *floR*	Absolute abundance enriched	Zhang et al. (2019a)
Activated carbon	15 g/L	Sludge and food waste	Mesophilic semi- continuous CoAD for 30 days	*sul1*, *sul2*, *tetA*, *tetW*, *tetO*, *tetX*, *tetB*, *tetM*, *tetQ*, *cmlA*, *floR*	Relative abundance enriched except for *tetQ* and *tetW*	Zhang et al. (2018a)
Activated carbon	5 g/L	Sewage sludge	Batch test for 10 days spiked with 25 mg/L sulfamethoxazole	*sul1*, *sul2*, *sul3*, *sul4*, *tetA*, *tetC*	Absolute abundance of ARGs decreased by 62%	Zhao et al. (2022)
Zero-valent iron					Absolute abundance of ARGs decreased by 48%	
Magnetite					Absolute abundance of ARGs decreased by 26%	
Coal gasification slag	5 and 10 g/L	Swine manure and wheat straw	Batch tests with TS of 8% at mesophilic CoAD for 50 days	*tetC*, *tetG*, *tetW*, *tetX*, *sul1*, *sul2*, *dfrA7*, *ermF*, *ermQ*, *ermX*, *qnrS*, *qnrA*, *intl1*, *intl2*, *Tn916/1545*, *ISCR*	*dfrA7*, *sul2*, *tetW*, *ermF*, *ermQ* were removed 25–90% after AD at 10 g/L *ISCR1* removal was 95%	Liu et al. (2019)
Graphite	Suspension stock of 50 mg/l	Sewage sludge and food waste	Batch thermophilic CoAD for 18 days	*sul1*, *sul2*, *tetM*, *tetO*, *tetQ*, *tetW*, *bla*_*OXA-1*_, *ermB*, *ermF*, *mefA*	Graphene improved removal of *bla*_*OXA-1*_, *ermF*, *ermB*, *tetQ*, *tetX* while GO improved *sul1*, *sul2*, *intl1*, *tetM*, *tetO*, *tetW*	Wang et al. (2021a)
Graphene						
graphene oxide (GO)						
Graphene oxide	100 and 800 mg/L	Swine manure and wheat straw	Batch mesophilic CoAD spiked with 227 mg/L copper for 50 days	*sul1*, *sul2*, *drfA7*, *tetC*, *tetG*, *tetM*, *tetW*, *tetX*, *aac(6′)-lb-cr*, *qnrA*, *qnrS*, *ermF*, *ermQ*, *ermX*, *bla*_*VIM*_, *bla*_*CTX-M*_	Lower GO concentration performed better in removal. Total absolute abundance reduction from 36 to 47%	Zhang et al. (2019g)
Biochar	5, 10, 15, 20% on TS basis	Swine manure	Batch mesophilic AD with TS 8% for 60 days	*sul1*, *sul2*, *parC*, *aac(6′)-lb-cr*, *ermB*, *ermF*, *tetW*, *tetG*, *tetX*, *bla*_*CTX-M*_, *bla*_*TEM*_, *aac(6′)-II*, *aadA1*	Total relative abundance reduced by 4–15% Abundance of *intl1*, *parC*, *tetX*, *bla*_*CTX-M*_, *bla*_*TEM*_, *aac(6′)-lb-cr*, *ermB*, *tetW* reduced significantly in 5% biochar	Yang et al. (2021)
Biochar	15 g/L	Swine manure	Batch mesophilic AD spiked with 0.5 and 50 mg/L tetracycline for 24 days	*tetA*, *tetG*, *tetC*, *tetM*, *tetO*, *tetT*, *tetX*	Absolute abundance reduced by 62–91%	Wang et al. (2022a)
Biochar	4 g/L	Swine manure and sewage sludge	CoAD in semi-continuous anaerobic sludge blanket at mesophilic condition with TS of 5 and 14% for 32 days	All known ARGs	Total ARG abundance decreased by 35%	Xu et al. (2022)
Biochar	20 g/L	Swine wastewater	Batch mesophilic AD for 45 days	*sul1*, *sul2*, *tetM*, *tetX*, *tetA*, *tetC*, *ermF*, *ermX*, *ermB*, *ampC-01*, *bla*_*CTX-M01*_, *bla*_*TEM*_	Total relative abundance declined by 23%	Wang et al. (2022b)
Nanoscale zero-valent iron	80 and 160 mg/L	Cattle manure	Batch mesophilic AD with TS of 8% for 43 days	*tetC*, *tetG*, *tetW*, *tetM*, *tetX*, *sul1*, *sul2*, *dfrA7*, *ermF*, *ermQ*, *ermX*, *gryA*, *aac(6′)-lb-cr*, *bla*_*VIM*_, *Tn916/1545*	Absolute abundance decreased by 5–9 logs at 160 mg/L	Ma et al. (2019)
Nanoscale zero-valent iron	0.5, 1, 2, and 4 g/L	Sewage sludge	Semi-continuous mesophilic AD with TS of 8% for 20 days	*tetW*, *tetT*, *tetE*, *ermF*, *ermT*, *ermA*, *sul1*, *sul2*, *bla*_*OXA*_, *dfrA12*, *aac(6′)-IB*	Total absolute abundance declined by 28–62%	Zhang et al. (2020d)
Magnetite nanoparticles					Total absolute abundance declined by 37–70%	
Zero-valent iron	15 g/L	Swine manure	Batch mesophilic and thermophilic AD with TS of 10% for 23 days	*sul1*, *sul2*, *ermB*, *ermF*, *bla*_*CTX-M*_, *bla*_*TEM*_, *tetM*, *tetG*, *tetX*, *mcr-1*	Relative abundance increased by 33%. All ARGs were reduced effectively except *sul2* and *tetM*	Zhang et al. (2018b)
Zero-valent iron	5, 75, 150, and 350 mmol	Swine manure	Batch mesophilic AD with TS of 5% for 56 days	124 ARGs	Antibiotic inactivation of aminoglycoside genes and antibiotic target production of tetracycline genes reduced by 25%	Zhang et al. (2021a)
Zero-valent iron	5 and 60 g/L	Sludge and kitchen waste	Semi-continuous thermophilic CoAD with 1:3 ratio (sludge: kitchen waste) on VSS basis for 24 days	*tetA*, *tetC*, *tetG*, *tetM*, *tetO*, *tetW*, *tetX*	Absolute abundance reduced by 1.4–3.9 logs	Gao et al. (2017)
Magnetite	5, 75, 150, and 350 mmol	Swine manure	Batch mesophilic AD with TS of 8% for 30 days	*sul1*, *sul2*, *ermB*, *ermF*, *ereA*, *mefA*, *bla*_*CTX-M*_, *bla*_*TEM*_, *tetM*, *tetG*, *tetX*, *mcr-1*	Did not influence total ARG abundance after AD	Zhang et al. (2019d)
Magnetite nanoparticles	5, 75, 150, and 350 mmol	Swine manure	Batch mesophilic AD with TS of 8% for 30 days	*sul1*, *sul2*, *ermB*, *ermF*, *ereA*, *mefA*, *bla*_*CTX-M*_, *bla*_*TEM*_, *tetM*, *tetG*, *tetX*, *mcr-1*	Relative abundance of most genes increased	Zhang et al. (2019e)

#### Carbon-based materials

Carbon-based additives include different types of materials like activated carbon and graphene. Generally, they have been used in different environmental applications due to their high porosity and surface area (Romero-Güiza et al. 2016). In AD, carbonaceous additives are utilized to reduce the effect of organic loading and enhance microbial growth. Enrichment of microorganisms during AD has been achieved through direct interspecies electron transfer (DIET) using conductive materials like activated carbon and graphene. The basis of this phenomenon is that the conductive materials act as a bridge (electron shuttle) to facilitate electron transfer in microorganisms (Yang et al. 2021; Deng et al. 2022).

Activated carbon (AC) has been widely utilized in many water and wastewater treatment methods on either lab or industrial scales. Several studies have investigated the effect of activated carbon (AC) on the fate of ARGs during different AD processes. It is speculated that AC could enhance microbial activity and growth in the AD system, probably due to its retention sites (Chen et al. 2016). Zhang et al. (2019a) reported that adding powdered activated carbon enhanced the attenuation of ARGs in the mono-digestion of food waste. However, it did not show a significant effect on ARGs in the co-digestion of food waste and chicken manure mainly because chicken manure may have contained antibiotics. Additionally, the author reported that AC caused a shift in the microbial community, specifically in archaeal species. In another study, Zhang et al. (2018a) revealed that digesters blended with AC could remove *tetQ* and *tetW* genes while the abundance of *tetA*, *tetM*, *tetO*, *sul1*, *sul2*, *cmlA*, and *floR* genes enriched after the process. A possible reason for the better efficiency of AC facilitated-AD processes in the removal of ARGs is that since AC possesses numerous retention sites, it can accommodate bacterial communities resulting in higher bacterial activity and less mobility. In other words, AC could reduce bacterial mobility because of its porous structure and large surface area which decrease microorganisms’ contact and results in a limited exchange rate of genetic materials like *intI1*, which decreases HGT in the AD system (Gyles and Boerlin 2013).

In addition to AC, coal gasification slag (CGS) and graphene oxide (GO) are other carbonaceous materials tested for their efficiency in removing ARGs. CGS includes residual carbon and inorganic elements shaped irregularly together and make a porous structure (Wu et al. 2007). Liu et al. (2019) utilized CGS as an additive for AD of swine manure to investigate the fate of five ARGs. They reported that the abundance of *dfrA7*, *sul2*, *tetW*, *ermF*, and *ermQ* reduced by 24–90% after AD at CGS 10 g/L. Also, the abundance of ISCR1 as MGE decreased after the process. The porosity and retention sites of CGS were attributed to the reduction of ISCR1, while the removal of potential bacterial hosts was considered the possible reason for the reduction of ARGs.

GO is another carbon-based material that is known for its exceptional properties. GO, especially its nanosheets, have abundant oxygen-containing groups such as hydroxyl, epoxide, and carboxyl, improving its reactivity. In a study, Zhang et al. (2019g) studied the effect of GO on AD of swine manure polluted by copper. They found that GO could facilitate the removal of ARGs in the system. The authors proposed four main reasons for this removal. First, GO’s large surface area makes it an excellent adsorbent that could decrease the selective pressure on microorganisms. Another reason is the oxygen-containing groups, and the p-boning system in GO chemically that binds with ARGs and limit their mobility. The third reason can be attributed to the removal of bacterial hosts that decreased the abundance of ARGs. Eventually, like other carbon-based adsorbents, GO could decrease MGEs leading to limited HGT in the system. In another study, Wang et al. (2021a) compared the effect of graphite, graphene, and GO on the removal of ARGs in co-digestion of SS and FW. The results showed that graphene had the best efficiency on *bla*_*OXA-1*_, *ermF*, *ermB*, *tetQ*, and tetX. However, GO showed better performance in the attenuation of *sul1*, *sul2*, *intl1*, *tetM*, *tetO*, and *tetW.*

Biochar is also another carbon-based material that has been studied broadly in AD processes to remove ARGs. Essentially, biochar is produced through organic waste pyrolysis and, in contrast to AC, is not activated. Recently, Wang et al. (2022a) investigated the effect of biochar under different tetracycline pressures (0.5 and 50 mg/L) on ARG removal. The authors reported that the addition of biochar could enhance ARGs’ abundance removal even at the high tetracycline concentration. Furthermore, the results showed that biochar dramatically limited enrichment of *Firmicutes* which were found to be correlated with ARGs linked to antibiotic target protection and antibiotic inactivation. In another study, Wang et al. (2022b) reported that the integration of biochar produced from dewatered swine manure could enhance total relative abundance of ARGs and mobile genetic elements by 23.5% and 74.8%, respectively. The possible reason for this attenuation was attributed to the immobility of bacterial communities and inhibition of potential hosts of ARGs due to the addition of biochar.

Yang et al. (2021) also reported that biochar containing system could remove *parC*, *tetX*, *bla*_*CTX*-M_, and *bla*_*TEM*_ with over 85% efficiency and *aac(6′)-Ib-cr*, *ermB*, and *tetW* with 20–40% removal efficiency. The absolute abundance declined after the addition of biochar. Similarly, the relative abundance of targeted genes declined after AD. In addition, the removal efficiency of *intl1* increased by 15% at a biochar dose of 5% (based on TS). Similar to AC, biochar also offers surface area to limit microbial mobility, therefore, limiting HGT. Also, Sun et al. (2018) investigated the impact of different biochar doses on ARG removal. They reported that while 5 g/L of biochar reduced the relative abundance of 5 out of 13 ARGs, 20 g/L dose could significantly reduce the total relative abundance of ARGs. Nonetheless, increasing the dosage to 50 g/L significantly increased the total relative abundance which was attributed to the presence of heavy metals in the biochar and co-selection of ARGs. Moreover, the results demonstrated that biochar could limit relative abundances of *Firmicutes* and *Proteobacteria* as potential hosts of ARGs.

#### Iron-based materials

Recently, different iron-based materials, including iron oxide, iron salts, and zero-valent iron (ZVI), have been added to AD to enhance methane yield and sulfate control. Additionally, some studies have investigated the effect of these materials on the fate of ARGs during the AD system. ZVI and nanoscale zero-valent iron (nZVI) have been reported to remove ARGs effectively, specifically tetracycline and beta-lactam genes (Gao et al. 2017; Wang et al. 2019c; Xiang et al. 2019; Zhou et al. 2021). Furthermore, it was reported that ZVI could facilitate hydrolysis-acidification reaction in AD, causing enhanced biological activities. Subsequently, extracellular DNA can be destroyed due to hydrolysis and biodegradation (Ma et al. 2011; Feng et al. 2014). Moreover, due to their cytotoxicity, nZVI and ZVI damage key cell membrane components, including functional proteins, through their corrosion products and consequently decrease the abundance of ARBs (Zhu et al. 2014). Besides, the removal of antibiotics like amoxicillin, ampicillin, tetracycline, and metronidazole was enhanced by nZVI, which decreases the selective pressure on microorganisms (Ghauch et al. 2009; Chen et al. 2012; Wang et al. 2016a). Zhang et al. (2021a) reported that ARG attenuation was dependent on ZVI dose. The removal efficiency improved from 4.7 to 25% when the ZVI dose increased from 5 to 75 mmol. Additionally, they inferred that ZVI addition mainly targeted tetracycline and aminoglycoside resistance genes. Moreover, *tnpA* was found to be the dominant MGE after AD which was attributed to the fact that the addition of ZVI facilitated DIET and microbial communication which increased HGT potential in AD.

Although magnetite (Fe_3_O_4_) and nanoscale magnetite have been reported to enhance methane production, their efficacy in removing ARGs has been demonstrated to be limited. Potential host elimination plays a vital role in the fate of ARGs during the AD process, and it was reported that magnetite-based materials could not significantly affect the microbial community (Zhang et al. 2019e). Zhang et al. (2019a) also investigated the effect of magnetite (5, 75, 150, and 350 mmol) on the fate of ARGs during AD of swine manure. The results showed that the significant effect of magnetite was observed on day 13 of the process so that as the concentration of magnetite increased, the total relative abundances of ARGs increased. On day 13, relative abundances of *ermF*, *tetX*, and *tetM* enriched while it reduced for *ereA* and *ermB*. At the end of the process (day 30), ARG abundance did not change significantly, but the relative abundance of *mefA* reduced noticeably. Xiang et al. (2019) reported that HGT increased after adding magnetite nanoparticles, probably due to excessive electron transfer. Other than ZVI and magnetite, the effects of other iron-based materials were tested on the fate of ARGs during the AD process. Also, Lu et al. (2020) investigated the effect of ferrous chloride (FeCl_2_) on the fate of ARGs in AD of swine manure. The scholars reported that the utilization of ferrous chloride enhanced methane production and promoted ARG removal. However, the detailed mechanisms remained unknown. The removal of ARGs in the study could be attributed to the bacterial community change as FeCl_2_ enhanced hydrogen utilization and DIET in microorganisms which affected the bacterial community. Zhang et al. (2020d) compared the effect of magnetite nanoparticles and nZVI on ARG attenuation in AD of sludge. They reported that both iron nanoparticles could significantly reduce total ARG absolute abundances. Moreover, an inverse correlation was found between iron nanoparticle dosage and total ARG removal. Also, tetracycline efflux and ribosomal protection resistance mechanisms were found to be disabled by both iron nanoparticles.

### Effect of operational parameters

The performance of an AD system is closely related to its operational conditions, and operating parameters play a crucial role in the system's efficiency. Temperature and digestion period are the two main parameters affecting an AD system. This section revolves around the effect of operating parameters on the fate of ARGs. Table 6 summarizes the effects of different operating parameters on the fate of ARGs.

**Table 6:** Effect of different operational parameters on ARGs fate in anaerobic digestion processes

Sample type	Operating conditions	Target genes	ARG dynamics	Reference
Chicken manure	Thermophilic AD at 60 °C for 30 days	*ampC*, *ermB*, *ermC*, *mecA*, *tetK*, *tetM*, *sulI*, *sulII*	All genes decreased as well as pathogenic bacterial groups	Anjum et al. (2017)
Waste activated sludge	Thermophilic AD at 55 °C and mesophilic AD at 35 °C	*sulI*, *sulII*, *tetA*, *tetO*, *tetX*, *bla*_*TEM*_, *bla*_*SHV*_	A declining trend in eARGs quantity was achieved. Thermophilic AD outperformed mesophilic AD	Zou et al. (2020a)
Activated sludge	Thermophilic AD at 55 °C and mesophilic AD at 35 °C	35 ARG types	Mesophilic AD outperformed thermophilic AD. *aadA*, *macB*, and *sul1* enriched in thermophilic AD. *sulI and tetM* enriched in mesophilic AD	Zhang et al. (2015)
Pig manure	Thermophilic AD at 55 °C and mesophilic AD at 35 °C for 25 days. Then, H_2_ was added to reactors	5 drug classes	Addition of H_2_ did not affect mesophilic AD, but in thermophilic AD, increased number of macrolides, glycopeptide, lincosamide, and fluoroquinolone genes	Zhu et al. (2021b)
Sludge	Psychrophilic AD at 15 °C and mesophilic AD at 35 °C for 35 days	14 ARG types	Psychrophilic AD performed better in the absence of oxytetracycline	Yun et al. (2021)
Cattle manure	Solid-state thermophilic AD at 55 °C, mesophilic AD at 35 °C and liquid AD at 35 °C	20 ARG types	Mesophilic solid-state achieved most reduction than other treatments	Sun et al. (2019c)
Sewage sludge	Mesophilic AD for 60 and 109 days	11 ARG types	Shorter SRT improved ARGs reduction in single-stage and two-stage digestion	Zhang et al. (2019b)
Sewage sludge	Mesophilic and thermophilic AD at different SRTs of 20, 30, and 40 days	*Sul1*, *sul2*, *tetW*, *tetX*, *tetA*, *ermF*, *aac(6′)-lb*, *intl1*, *intl2*, *tnpA*	Higher removal of ARGs was achieved in long SRTs in mesophilic AD while thermophilic AD removed more ARGs in short SRTs	Mortezaei et al. (2023)

#### Temperature

Based on temperature, AD can be classified into psychrophilic (15–20 °C), mesophilic (30–38 °C), and thermophilic (50–57 °C) conditions (Lettinga et al. 2001; Appels et al. 2008). Temperature is an essential parameter in AD processes because it influences microbial community and consequently ARG fate (Lin et al. 2016). The results of the effect of different temperatures on the fate of ARGs are widely varying. On the one hand, several studies have reported that thermophilic conditions could enhance the removal of ARGs in AD systems (Diehl and LaPara 2010; Anjum et al. 2017; Min Jang et al. 2019; Wen et al. 2020). The main reason for this reduction during thermophilic AD is the change in diversity and abundance of the microbial community in the system. The potential hosts for ARGs have been reported to be eliminated under high temperatures (Zou et al. 2020a). Tian et al. (2019) revealed that most organisms from primary and secondary sludge are resistant bacteria of gut and activated sludge that are mesophilic or aerobic. Thus, they cannot resist high temperatures resulting in the release of their DNA which fermenting bacteria will degrade them. Also, they reported that high temperatures could destroy MGEs (*intl1*, *TP614*, *tnpA*, *IS613*) in the AD system giving rise to HGT limitation. In another study, Zou et al. (2020b) investigated the fate of extracellular ARGs (eARGs) during thermophilic and mesophilic AD of sludge. They observed higher removal in the thermophilic condition. The main reason was due to the reduction of total suspended solids (TSS) and sludge extracellular polymeric substances (EPS) because they were the main locations for the accommodation of extracellular DNA (eDNA), and loss of them could expose eDNA to extracellular DNases, which can degrade eDNA and eARGs.

On the other hand, some other studies reported that the ARG abundance increased during thermophilic AD (Zhang et al. 2015; Huang et al. 2019; Sun et al. 2019c). Several reasons, including different substrate and operational conditions, and enhanced ARB activity at thermophilic AD were considered for the variation in results. For instance, Huang et al. (2019) reported that higher temperatures promoted microbial activity, especially pathogenic bacteria such as *Streptococcus*. Additionally, it was reported that the integrase genes were enriched. Hence, it increases the chance of HGT during thermophilic digestion. In another study, Flores-Orozco et al. (2022) showed that the substrate type could affect the ARG attenuation significantly as thermophilic AD was found to be effective for ARG removal in pig manure rather than cattle manure.

In addition to mesophilic and thermophilic AD, Yun et al. (2021) investigated the effect of psychrophilic AD of sludge on the fate of ARGs and compared it with the mesophilic condition. The researchers revealed that the psychrophilic condition could reduce the abundance of dominant genera while the community richness was kept constant. In addition, bacterial richness was negatively correlated with tetracycline ARGs compared to other ARG types. They also reported that the effect of temperature was complex, and the higher temperature did not necessarily result in a better reduction of ARGs. In turn, if the ARG co-selectors like heavy metals and antibiotics were removed from sludge, the lower temperature would benefit ARG removal. Nonetheless, further studies are required to deeply investigate the effect of low temperatures on the fate of ARGs in the AD process. These contradictory results accentuate the necessity for future studies that can deeply investigate the effect of various temperatures on microbial community and mechanisms of ARG removal in order to find the optimal temperature for ARG removal.

#### Digestion time

Solid retention time (SRT) or the digestion period is an important operational parameter in AD systems because it can affect microbial communities. However, few investigations have been done on the effect of SRT on the fate of ARGs in AD systems. Ma et al. (2011) investigated the fate of ARGs during AD of sludge at different digestion periods. They revealed that longer SRT had better efficiency in removing *sul1*, *sulII*, and *tetG*. However, in both SRTs, similar types of ARGs increased or decreased. In another study, Zhang et al. (2019a) studied single-stage AD, microwave pretreatment + AD, and microwave pretreatment + two-stage AD of raw sewage sludge. The results showed that decreasing SRT from 20 to 15 days could facilitate the reduction of ARGs in single-stage AD of raw residual sewage sludge and two-stage digestion of microwave-pretreated sewage sludge. However, the removal of ARGs during single-stage AD of microwave-pretreated sludge could benefit from longer SRT. According to the results, macrolide resistance genes could be eliminated in shorter SRT, but longer SRT was required to eliminate sulfonamides and tetracycline resistance genes. Recently, Mortezaei et al. (2023) studied the effect of temperature and SRT on the attenuation of ARGs and MGEs. The results showed that thermophilic AD outperformed (75.8% removal efficiency) mesophilic AD in short SRTs (20 days) whereas in longer SRTs (30 and 40 days) mesophilic AD performed better than thermophilic AD. However, it is not clear how SRT could affect the bacterial communities and ARG host in these two different temperatures. Thus, further investigations that study a wide range of SRTs and analyze ARGs hosts are required with respect to the above studies.

#### Miscellaneous operational conditions

In addition to common operating parameters such as temperature and SRT, other studies looked into the effects of other operational conditions or configurations that could affect the attenuation of ARGs in AD systems. In this section, these studies are discussed. Different startup methods can affect the performance of AD systems and, subsequently, the fate of ARGs during AD. However, limited information is available on the effect of starting modes. Recently, Zhi et al. (2021) studied the effect of five different starting modes on the fate of various ARGs during high solids AD of pig manure. The starting modes were controlled without reflux spray (T1), leachate refluxing (T2), water flushing (T3), biogas slurry flushing (T4), and pH regulator flushing (T5). They observed that although all the techniques could reduce MGEs (*intl1*, *ISCR1*, *Tn916/1545*), the reduction trend for ARGs was different. *tetM*, *tetO*, *oqxB*, *fexA*, and aadA decreased in T1–T4 while increased in T5 probably because of the variation in the pH value of T5. Overall, system T4 was found to be the most efficient starting mode for ARG removal and high microbial diversity. *Firmicutes* phylum was found to play a critical role in transmitting ARGs. In a similar study, Gao et al. (2022) conducted research on the effects of starting modes on ARG removal in high solid AD. The starting modes were control (T1), self-produced leachate reflux (T2), water flush followed by leachate reflux (T3), flush with biogas slurry followed by leachate reflux (T4), and leachate reflux with pH regulation with NaOH solution (T5). The results showed that the main reduction of ARGs occurred in the acidification stage while T4 and T5 systems showed high microbial communities, diversity, and richness. Also, these two systems contained higher abundances of ARGs than other systems. On the other hand, during gas production and ending stages, bacterial richness and diversity became lower. Hence, more studies are needed to shed light on the mechanisms involved, particularly, at different stages of AD. Currently, there is a lack of knowledge on ARG fate in acidification or gas production stages.

Two-stage AD which includes one acidogenic phase followed by a methanogenic phase has been studied broadly for enhancement of AD performance. Recently, studies in addition to AD performance have also focused on the fate of ARGs in two-stage AD processes. Shi et al. (2021) reported that ARGs showed different trends between acidogenic and methanogenic stages. The results showed that macrolide, lincosamide, and streptogramin (MLS) resistance genes increased in the alkaline fermentation stage while they decreased in the methanogenic phase in both thermophilic and mesophilic conditions. An opposite trend was observed for tetracycline resistance genes. In the mesophilic condition, tetracycline genes increased in both stages while they reduced in the first stage of the thermophilic condition. Overall, two-stage AD showed higher efficiency in ARG attenuation. In another study, Jang et al. (2020) compared full-scale two-stage thermophilic AD with full-scale single-stage thermophilic AD in the removal of certain ARGs. The results showed that two-stage AD remarkably enhanced ARG attenuation even though it was performed in shorter residence time (15–17 days vs 39 days). In particular, *tetG*, *tetH*, *tetM*, *tetQ*, *tetX*, and *intl1* were completely removed in the two-stage AD. More recently, Damtie et al. (2022) compared different operational factors including temperature, configuration, SRT, and operation mode in AD of different substrates to investigate ARG removal. The results demonstrated that digesters’ configuration (single- vs two-stage) and other operating parameters have a higher effect on ARG abundances than substrate types. The highest removal efficiency (99.99%) was observed in two-stage thermophilic AD with a long residence time (32 days). The possible reasons for this phenomenon can be attributed to the fact that the acidic pH condition in the first stage is favorable for ARG reduction while the neutral phase in the methanogenic phase is more favorable for HGT. Additionally, long residence times can provide oligotrophic conditions in which the reproduction and diversity of ARBs will be limited.

## Current challenges and future perspectives

**Fig. 4:**
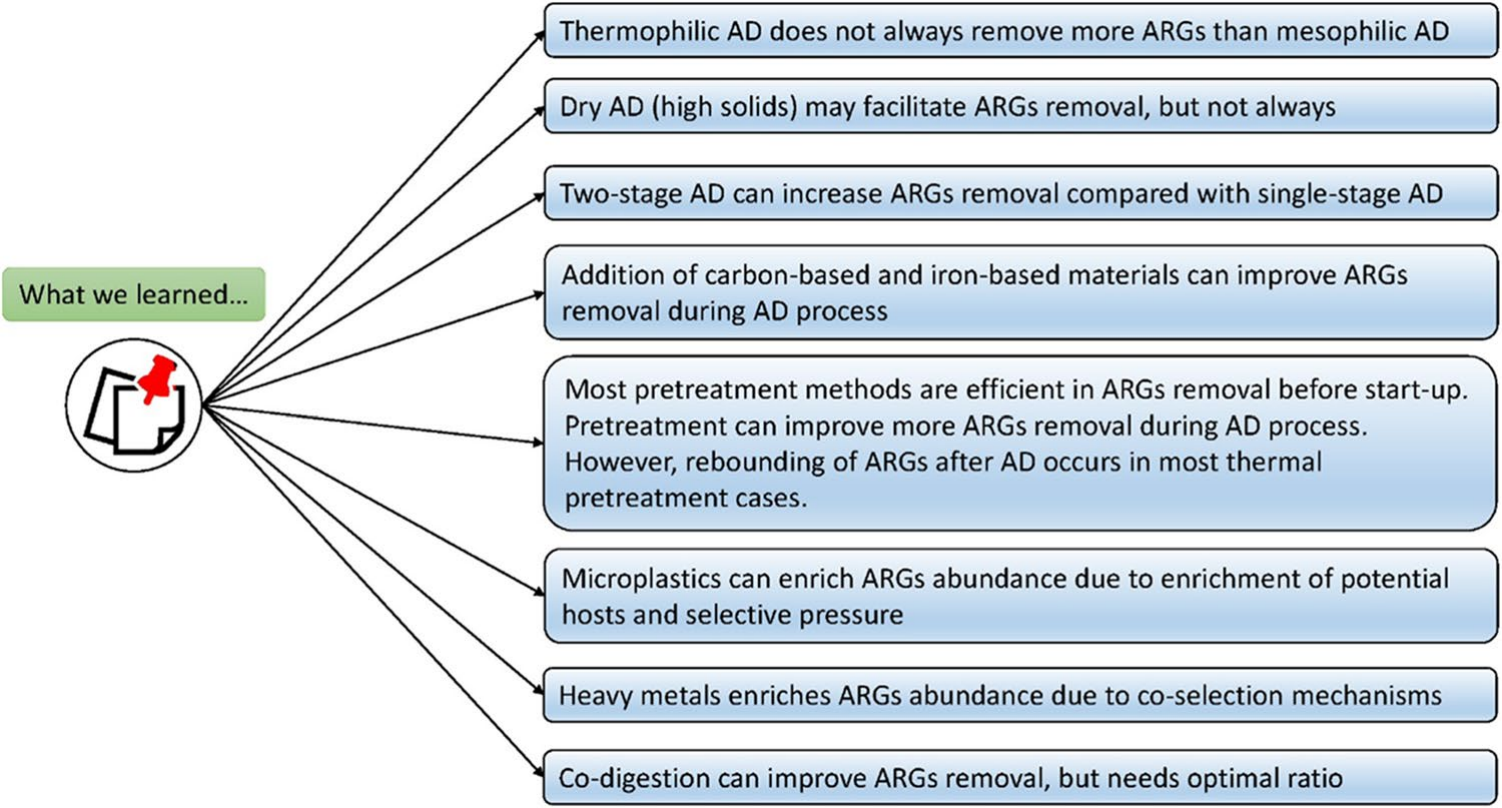
Summary of take-home messages of influencing factors on ARGs fate in AD process

The take-home messages of influencing factors are shown in Fig. 4. Over the past 10 years, several studies have been conducted on the fate of ARGs in the AD processes. However, there are numerous knowledge gaps regarding their fate in AD processes, and more studies are needed to shed light on the subject because generalization is challenging due to some contradictions in the current literature. As shown in Fig. 4, higher temperatures seem to be effective in the removal of ARGs in AD processes, but some studies did not find similar results which were discussed earlier. Based on most studies, high-solid AD could improve ARG removal, but since the number of studies on the fate of ARGs in high-solid AD is limited, more studies are required to deeply investigate the effects of solids on the fate of ARGs. Different operating conditions such as two-stage AD were also found to be beneficial for ARG removal. However, it is crucial to implement pilot to full-scale tests to investigate their efficacy.

Furthermore, with the growing trend in nanomaterials, the use of additives in AD processes has increased. Based on the current literature, the use of additives, in particular, conductive ones, could efficiently affect ARG propagation in AD processes. However, the characteristics of the material should be studied more to shed more light on the mechanisms involved in the fate of ARGs. Pretreatment methods such as thermal hydrolysis have been shown substantial effectiveness in ARG removal, but the rebounding effect is inevitable in most cases reported in the literature. The presence of different compounds and materials like microplastics and heavy metals in the substrate is shown to affect the fate of ARGs in the AD process. Nonetheless, it is still challenging to study the mechanisms governing such a phenomenon.

Additionally, recent studies have utilized common statistical and bioinformatic tools to study the correlation between ARGs and microbial community and environmental parameters. However, in the future, a hybrid system including novel metagenomics and traditional detection methods can help researchers fill knowledge gaps. Since the digestate is usually used as biosolids, it is imperative to investigate the efficiency of AD in the removal of ARGs. Hence, the following knowledge gaps are identified and recommended for investigations in future studies:

- Although some studies aimed to investigate the fate of ARGs in different AD feedstocks, most studies selected swine manure and sewage sludge with more focus on AD performance. Thus, more studies are needed to study the fate of ARGs in parallel across multiple feedstocks, or co-feedstocks, such as food waste, poultry manure, and cattle manure, during digestion.
- Several studies investigated the impact of feedstock characteristics on the fate of ARGs during AD systems. However, the main mechanisms governing the removal, transfer, or resistance of genes are still unknown. For instance, there is still limited knowledge on the role of microplastics or other emerging contaminants on the fate of ARGs in AD.
- Some studies investigated the presence of ARGs in different seasons and reported that seasonal variations could affect ARGs’ presence. However, limited data are available on the effect of seasonal changes on the fate of ARGs during AD.
- Regarding operational parameters, only temperature is well studied. More studies are required to compare different operational parameters such as starting modes and two-stage AD and investigate their effect on the eventual fate of ARGs in AD systems.
- The main mechanisms governing the transfer and hosts of ARGs in AD systems are still unclear. Standard statistical analyses have been applied to develop a correlation between ARGs and specific bacterial hosts. However, a combination system including culture methods and novel metagenomics should be implemented for future studies. Also, artificial intelligence and machine learning tools can help researchers to overcome these gaps.
- AD systems harbor a mixed and complex microbial community. However, the interaction between these communities regarding ARG fate and transfer is unclear. Future research should focus on evaluating the factors influencing gene transfer mechanisms and intricate quorum sensing between these communities in AD systems.

## Conclusion

This review summarized the occurrence and dissemination mechanisms of ARGs in the environment and discussed ARG fate during the AD process and provided an overview of the different parameters affecting their fate. Previous studies revealed that WWTPs and livestock manure are the main hotspots of ARGs in the environment. Pretreatment technologies showed promising results. However, future studies are required to overcome the rebound effect after the AD process. Literature reports showed that iron- and carbon-based materials can improve ARG removal along with biogas production and the development of new additive materials would be essential for future studies. Microbial community structure evolution and the influence of operational parameters seem to play a vital role in the fate of ARGs during AD. Future study objectives are identified and laid out which would entail understanding the underlying mechanisms of these factors on the fate of ARGs during the AD process.
